# Supplementary material for: Mouse models to investigate in situ cell fate decisions induced by p53
Source: EMBO J. 2024 Aug 19;43(19):12. doi: 10.1038/s44318-024-00189-z (PMC11445477; doi:10.1038/s44318-024-00189-z)
Supplement: Supplementary file 1 — Appendix [file 44318_2024_189_MOESM1_ESM.pdf]

Appendix for

Mouse models to investigate *in situ* cell fate decisions induced by p53

Elizabeth Lieschke *et al.*

*Corresponding author. Email: gkelly@wehi.edu.au; strasser@wehi.edu.au

This PDF file includes:

Appendix Figure S1……p2
Appendix Figure S2……p3
Appendix Figure S3……p4
Appendix Table S1……p5
Appendix Table S2……p6
Appendix Table S3……p7

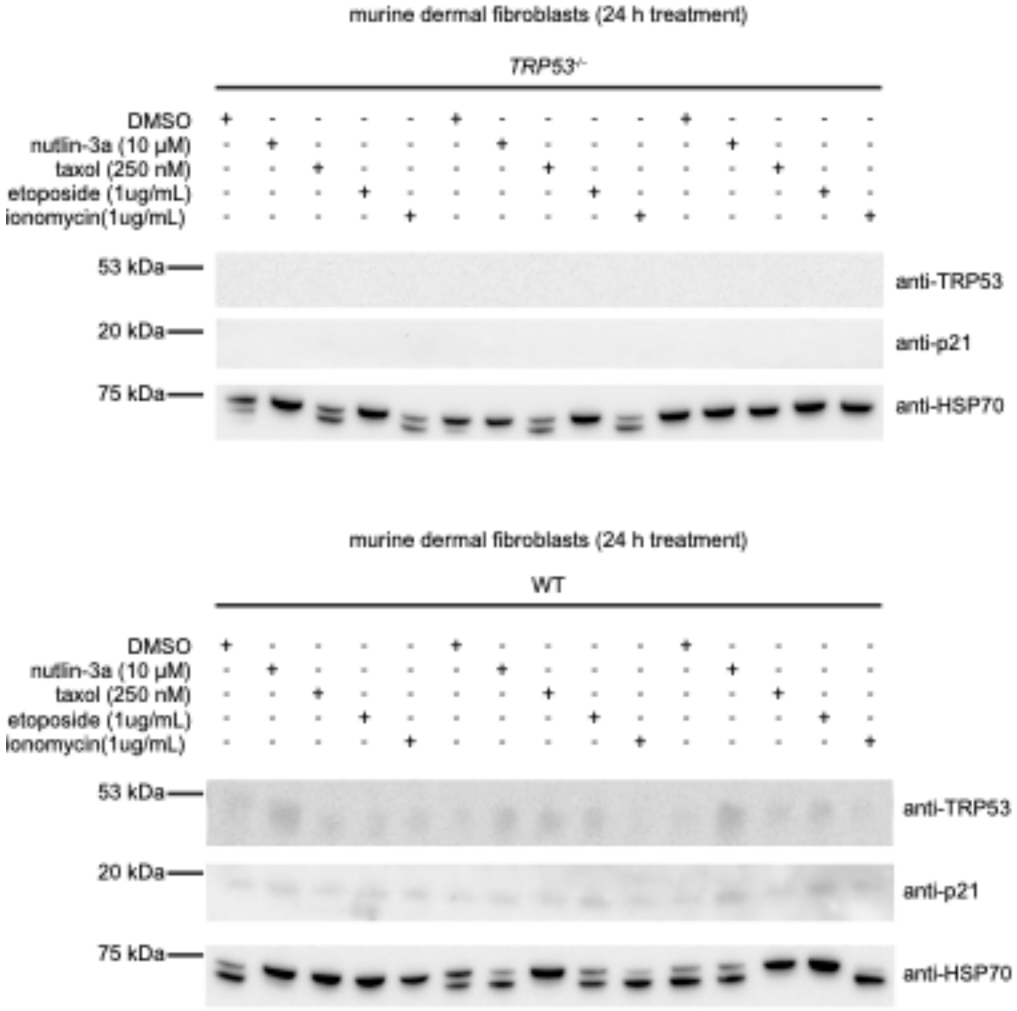

Appendix Figure S1. Extra data related to expression of p21 in fibroblasts. Top panel - Western Blot of *Trp53*$^{-/-}$ murine dermal fibroblasts. Both TRP53 and P21 expression is undetectable by western blotting in these cells. Bottom panel - Western Blot of wildtype murine dermal fibroblasts. Both TRP53 and P21 protein are detectable before and after treatment.

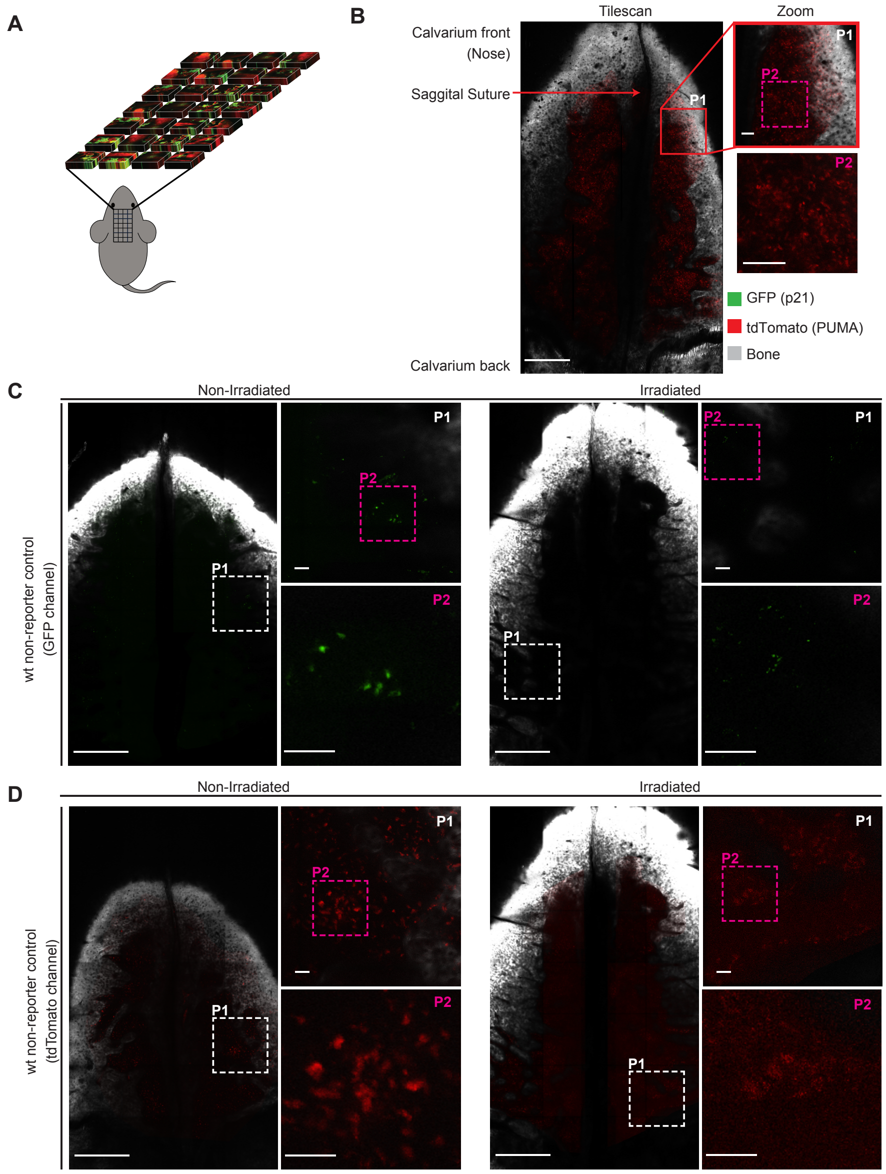

Appendix Figure S2. Extra data related to intravital imaging of calvarium. (**A**) Schematic showing location of imaging window in calvarium of the skull. A tile scan across the whole calvarium is collected at each time point. (**B**) Diagram showing orientation of the calvarium images and structural landmarks within the calvarium. (**C**) Images in the GFP channel of the calvarium from a wt mouse that had either received one dose of γ-irradiation (5 Gy) or left non-irradiated for 24 h. Little GFP signal is detected. Representative image in the tdTomato channel from n=3 mice. (**D**) Images of the calvarium from a wt mouse that had either received one dose of γ-irradiation (5

Gy) or left non-irradiated for 24 h. Little tdTomato signal is detected. Representative image from n=3 mice per genotype and treatment.

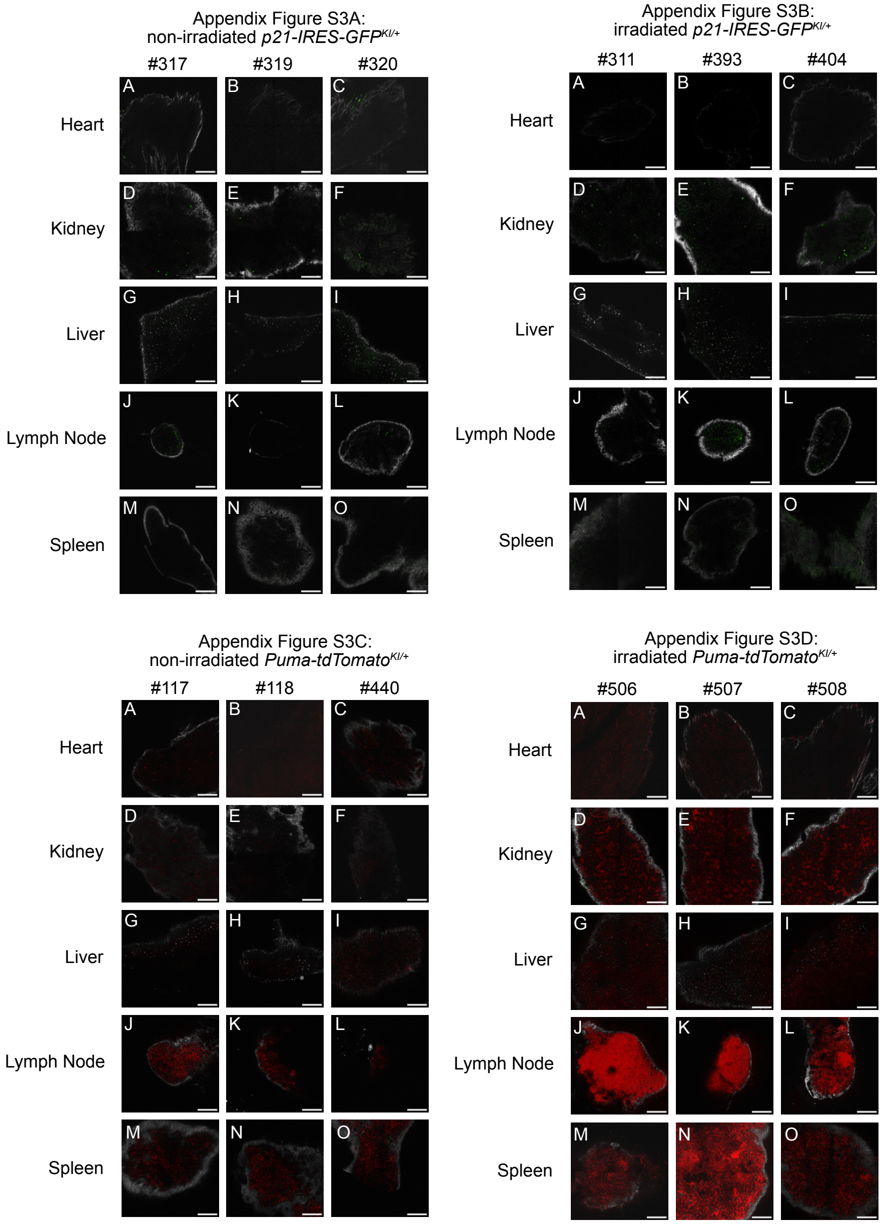

Appendix Figure S3. Extra data related to intravital imaging of multiple organs. Multiphoton microscopy images of heart, kidney, liver, lymph nodes, and spleen from three *p21-IRES-GFP*$^{KI/+}$mice (**B**) or *Puma-tdTomato*$^{KI/+}$ mice (**D**) 18 h after administration of a single dose of 5 Gy whole-body γ-irradiation. Organs from three non-irradiated *p21-IRS-GFP*$^{KI/+}$mice (**A**) or *Puma-tdTomato*$^{KI/+}$ mice (**C**) were used as controls. Images S3Aa, d, j, and S3Bc, e, k, are displayed in Figure 5. S3Ca, d, j, and S3Db, f, l are displayed. in Figure 8. Scale bars are 200 um.

Appendix Table S1. ***FLAG-Trp53*** **aging cohort summary information**

Mouse strain	mouse identifier	Genotype	Sex	Age at death (days)	Reason for death
FLAG-Trp53	88	KI/KI	F	451	End of experiment, no abnormalities
FLAG-Trp53	115	KI/KI	F	400	End of experiment, no abnormalities
FLAG-Trp53	116	KI/KI	F	400	End of experiment, no abnormalities
FLAG-Trp53	1	KI/KI	F	824	End of experiment, no abnormalities
FLAG-Trp53	125	KI/KI	F	533	End of experiment, no abnormalities
FLAG-Trp53	128	KI/KI	F	533	End of experiment, no abnormalities
FLAG-Trp53	133	KI/KI	F	522	End of experiment, no abnormalities
FLAG-Trp53	92	KI/KI	M	570	weight loss
FLAG-Trp53	93	KI/+	M	592	respiration increased, weight loss
FLAG-Trp53	17	KI/+	F	859	enlarged spleen, abnormal gait
FLAG-Trp53	42	KI/+	F	804	enlarged LN, increased respiration, intestines air filled, liver pale, abnormal gait
FLAG-Trp53	48	KI/+	F	799	milky fluid in peritoneum, air filled uterus, bones soft, muscle degeneration
FLAG-Trp53	49	KI/+	F	742	unhealed skin wound on back
FLAG-Trp53	59	KI/+	F	895	lumpy pancreas, milky fluid in peritoneum, increased resp, weight loss
FLAG-Trp53	104	KI/+	F	763	muscle degeneration, tumour on liver
C57BL/6	68	+/+	M	564	enlarged/bloody seminal vesicles
C57BL/6	70	+/+	M	786	Abdominal mass
C57BL/6	78	+/+	M	706	respiration increased
C57BL/6	75	+/+	M	460	anaemic
C57BL/6	76	+/+	M	681	Perineal hernia
C57BL/6	77	+/+	M	741	Abdominal mass, increased respiration
C57BL/6	66	+/+	M	739	Abdominal mass

Appendix Table S2. statistical analysis of DNA content analysis in *p21-IRES-GFP* mice

		wildtype			*p21-IRES-GFP* $^{KI/KI}$			*p21-IRES-GFP* $^{KI/+}$			*Trp53* $^{-/-}$*;p21-IRES-GFP* $^{KI/+}$		
		G0/G1	S	G2/M	G0/G1	S	G2/M	G0/G1	S	G2/M	G0/G1	S	G2/M
24 h	Abemaciclib	0.9997	>0.9999	0.5693	>0.9999	>0.9999	>0.9999	>0.9999	>0.9999	>0.9999	>0.9999	>0.9999	>0.9999
	Dexamethasone	>0.9999	>0.9999	>0.9999	>0.9999	>0.9999	>0.9999	0.6933	>0.9999	>0.9999	>0.9999	>0.9999	>0.9999
	Etoposide	0.8471	0.7866	0.6374	0.9974	0.3572	0.9115	**<0.0001**	0.3632	**<0.0001**	**<0.0001**	0.9694	**<0.0001**
	Nutlin-3a	>0.9999	0.445	>0.9999	>0.9999	0.9998	>0.9999	>0.9999	0.1158	0.6011	>0.9999	>0.9999	>0.9999
	Taxol	**<0.0001**	0.8447	**<0.0001**	**0.0047**	>0.9999	0.0777	**<0.0001**	**0.0115**	**<0.0001**	**<0.0001**	>0.9999	**0.0002**
	Thapsigargin	>0.9999	>0.9999	>0.9999	>0.9999	>0.9999	>0.9999	0.3756	>0.9999	0.6552	0.9514	>0.9999	>0.9999
48 h	Abemaciclib	0.6738	>0.9999	0.7856	>0.9999	>0.9999	0.9713	0.0535	>0.9999	**<0.0001**	>0.9999	>0.9999	>0.9999
	Dexamethasone	>0.9999	>0.9999	>0.9999	0.797	>0.9999	0.9922	>0.9999	>0.9999	>0.9999	>0.9999	>0.9999	>0.9999
	Etoposide	**<0.0001**	0.9996	**<0.0001**	**0.0328**	>0.9999	**0.0336**	**<0.0001**	0.9469	**<0.0001**	**0.0002**	>0.9999	**0.0005**
	Nutlin-3a	>0.9999	>0.9999	>0.9999	>0.9999	>0.9999	>0.9999	>0.9999	>0.9999	0.9351	>0.9999	>0.9999	>0.9999
	Taxol	**<0.0001**	0.9282	**<0.0001**	**<0.0001**	>0.9999	**0.0005**	**<0.0001**	**0.0431**	**<0.0001**	**0.0001**	>0.9999	**0.0002**
	Thapsigargin	0.9026	>0.9999	>0.9999	0.9996	>0.9999	0.9998	>0.9999	0.4777	>0.9999	>0.9999	>0.9999	0.9328
72 h	Abemaciclib	>0.9999	0.5008	0.0953	0.9708	>0.9999	0.1874	>0.9999	**0.0071**	0.6315	>0.9999	>0.9999	>0.9999
	Dexamethasone	>0.9999	>0.9999	>0.9999	>0.9999	>0.9999	0.9997	0.8543	>0.9999	0.6169	>0.9999	>0.9999	>0.9999
	Etoposide	**<0.0001**	>0.9999	**<0.0001**	**0.002**	>0.9999	**0.0002**	**<0.0001**	>0.9999	**<0.0001**	0.0602	>0.9999	0.0569
	Nutlin-3a	0.9915	>0.9999	>0.9999	>0.9999	>0.9999	>0.9999	**<0.0001**	>0.9999	**<0.0001**	>0.9999	>0.9999	>0.9999
	Taxol	**<0.0001**	>0.9999	**<0.0001**	**<0.0001**	>0.9999	**<0.0001**	**<0.0001**	>0.9999	**<0.0001**	**0.0007**	>0.9999	0.0608
	Thapsigargin	>0.9999	>0.9999	0.5767	>0.9999	>0.9999	>0.9999	>0.9999	>0.9999	>0.9999	>0.9999	>0.9999	>0.9999

Appendix Table S3. Statistical analysis of DNA content analysis in cells from *Puma-tdTomato* and *Trp53$^{-/-}$;Puma-tdTomato* mice.

		Puma-tdTomato $^{KI/+}$			*Trp53$^{-/-}$;Puma-tdTomato$^{KI/+}$*		
		G0/G1	S	G2/M	G0/G1	S	G2/M
24 h	Abemaciclib	**0.0296**	>0.9999	**0.0099**	>0.9999	0.3138	0.4118
	Etoposide	**<0.0001**	0.7924	**<0.0001**	**<0.0001**	0.9817	<0.0001
	Nutlin-3a	**<0.0001**	0.4737	**0.0052**	0.9996	>0.9999	>0.9999
	Palbociclib	0.9997	>0.9999	>0.9999	>0.9999	>0.9999	>0.9999
	Taxol	**<0.0001**	0.2293	**<0.0001**	**<0.0001**	0.578	<0.0001
48 h	Abemaciclib	**0.0361**	0.8462	**0.0004**	0.9846	>0.9999	0.615
	Etoposide	**<0.0001**	>0.9999	**<0.0001**	**<0.0001**	>0.9999	<0.0001
	Nutlin-3a	**<0.0001**	>0.9999	**0.0006**	>0.9999	>0.9999	>0.9999
	Palbociclib	>0.9999	>0.9999	>0.9999	>0.9999	>0.9999	0.9979
	Taxol	**<0.0001**	0.9989	**<0.0001**	**0.0002**	>0.9999	0.0003
72 h	Abemaciclib	0.9985	0.8289	0.1993	0.9963	>0.9999	0.9631
	Etoposide	**<0.0001**	>0.9999	**<0.0001**	**0.0132**	>0.9999	0.0266
	Nutlin-3a	**0.0048**	>0.9999	**0.0104**	>0.9999	>0.9999	>0.9999
	Palbociclib	**0.0301**	0.9975	0.3008	0.9235	>0.9999	0.995
	Taxol	**<0.0001**	>0.9999	**<0.0001**	0.0816	>0.9999	0.2077
