# Supplementary figures and images for: Mouse models to investigate in situ cell fate decisions induced by p53
Source: EMBO J. 2024 Aug 19;43(19):12. doi: 10.1038/s44318-024-00189-z (PMC11445477; doi:10.1038/s44318-024-00189-z)

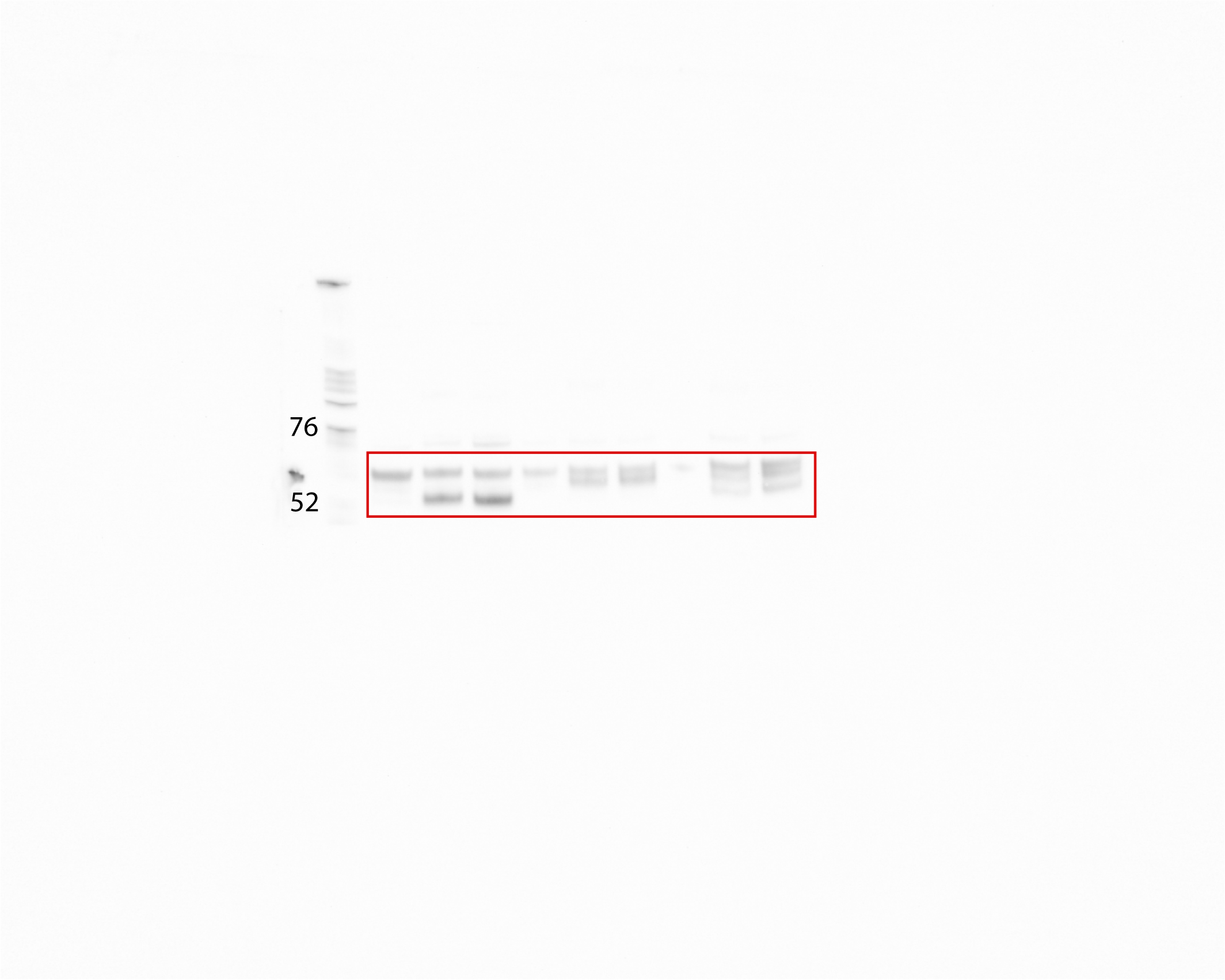

Supplement: Supplementary file 5 — Source data Fig. 2 [file 44318_2024_189_MOESM5_ESM.zip › Figure 2/2A/TRP53.tif]

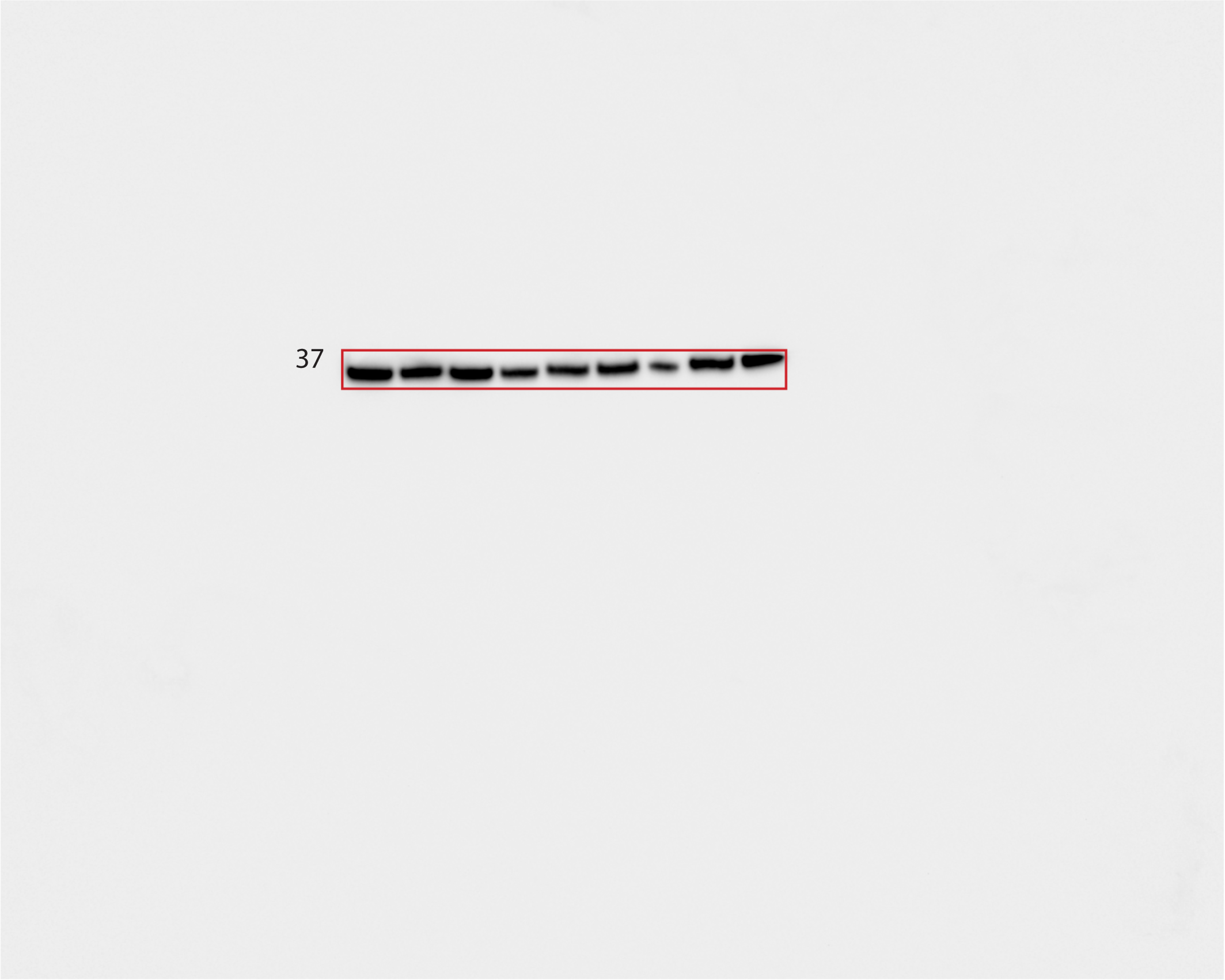

Supplement: Supplementary file 5 — Source data Fig. 2 [file 44318_2024_189_MOESM5_ESM.zip › Figure 2/2A/GADPH.tif]

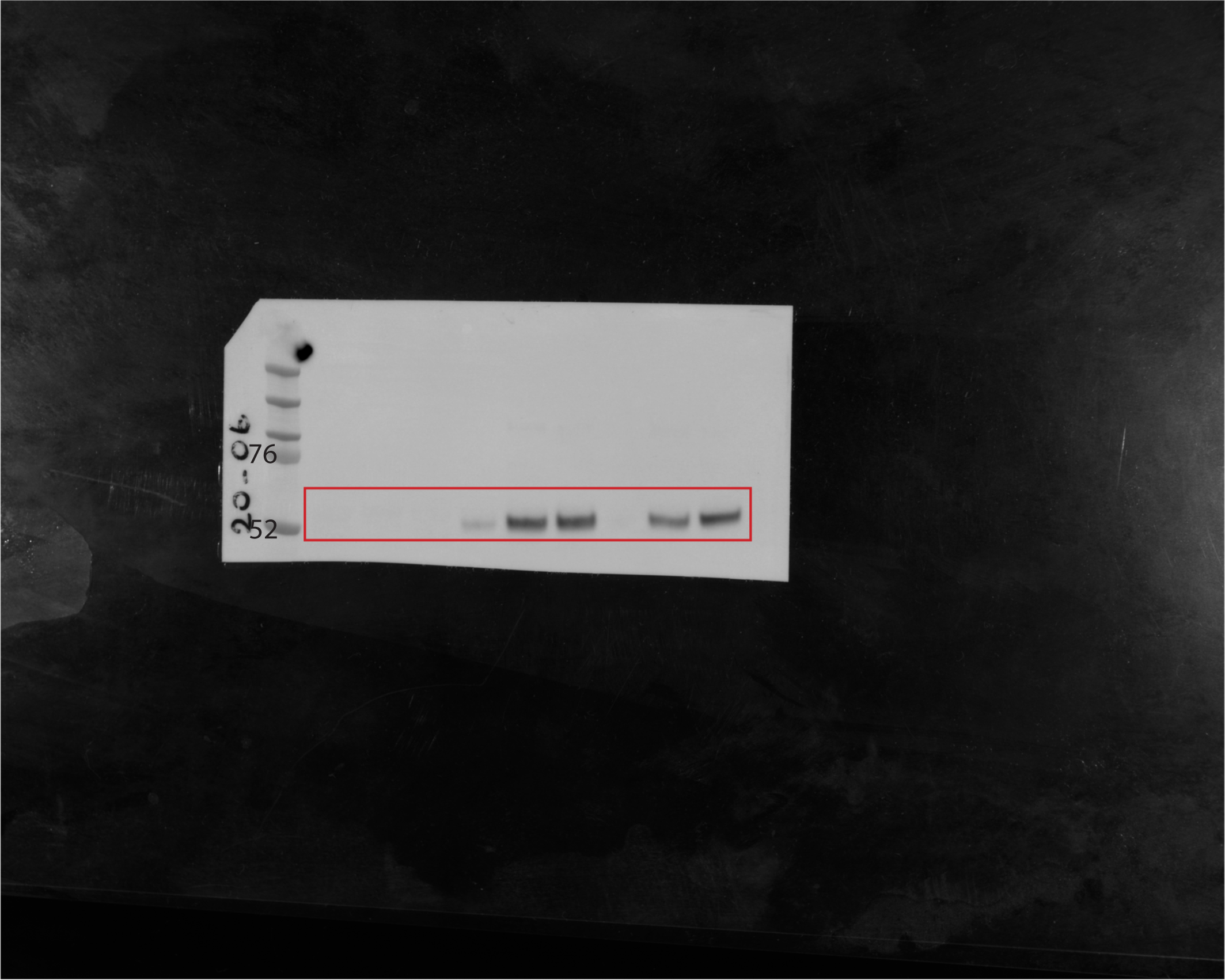

Supplement: Supplementary file 5 — Source data Fig. 2 [file 44318_2024_189_MOESM5_ESM.zip › Figure 2/2A/FLAG.tif]

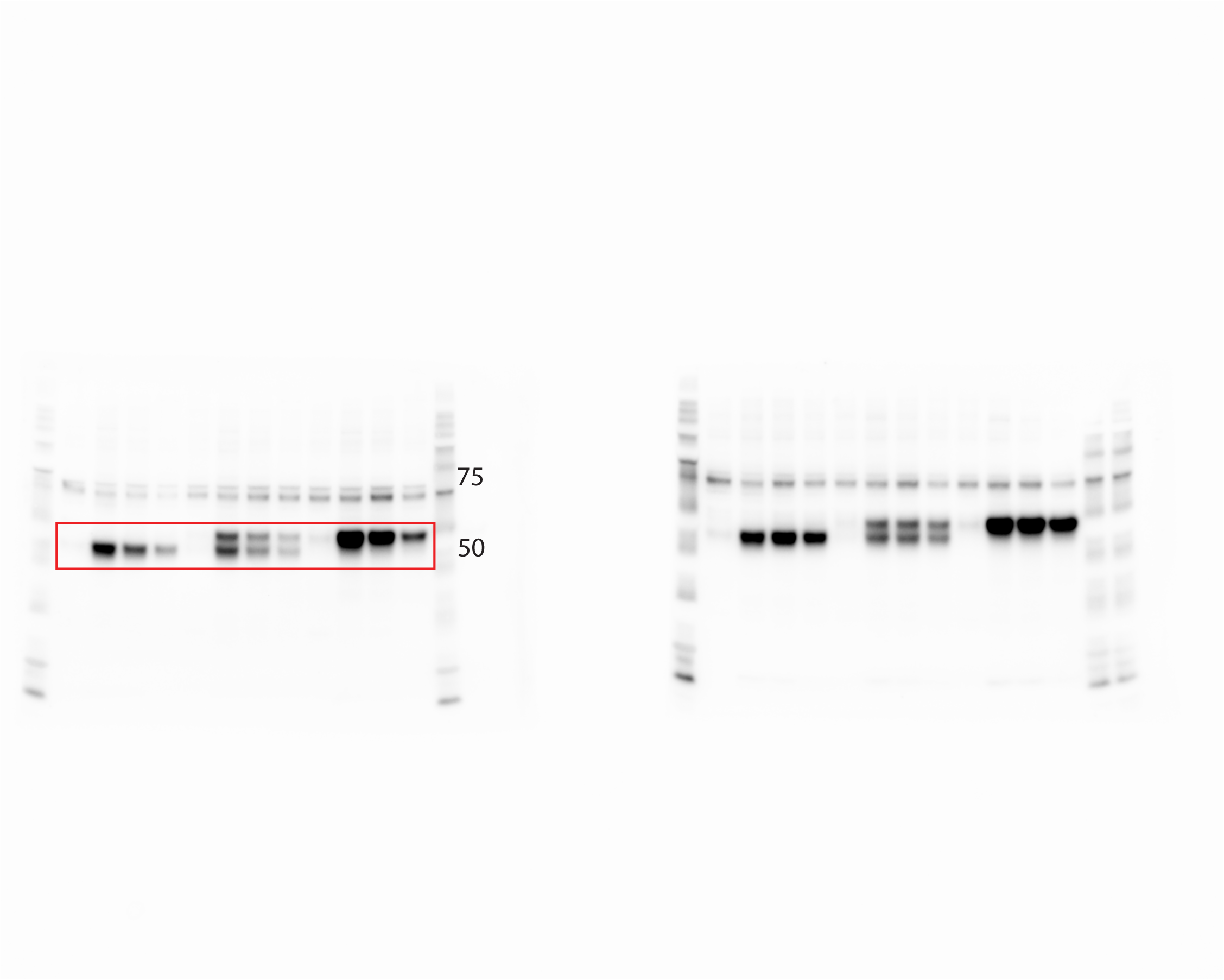

Supplement: Supplementary file 5 — Source data Fig. 2 [file 44318_2024_189_MOESM5_ESM.zip › Figure 2/2C/TRP53.tif]

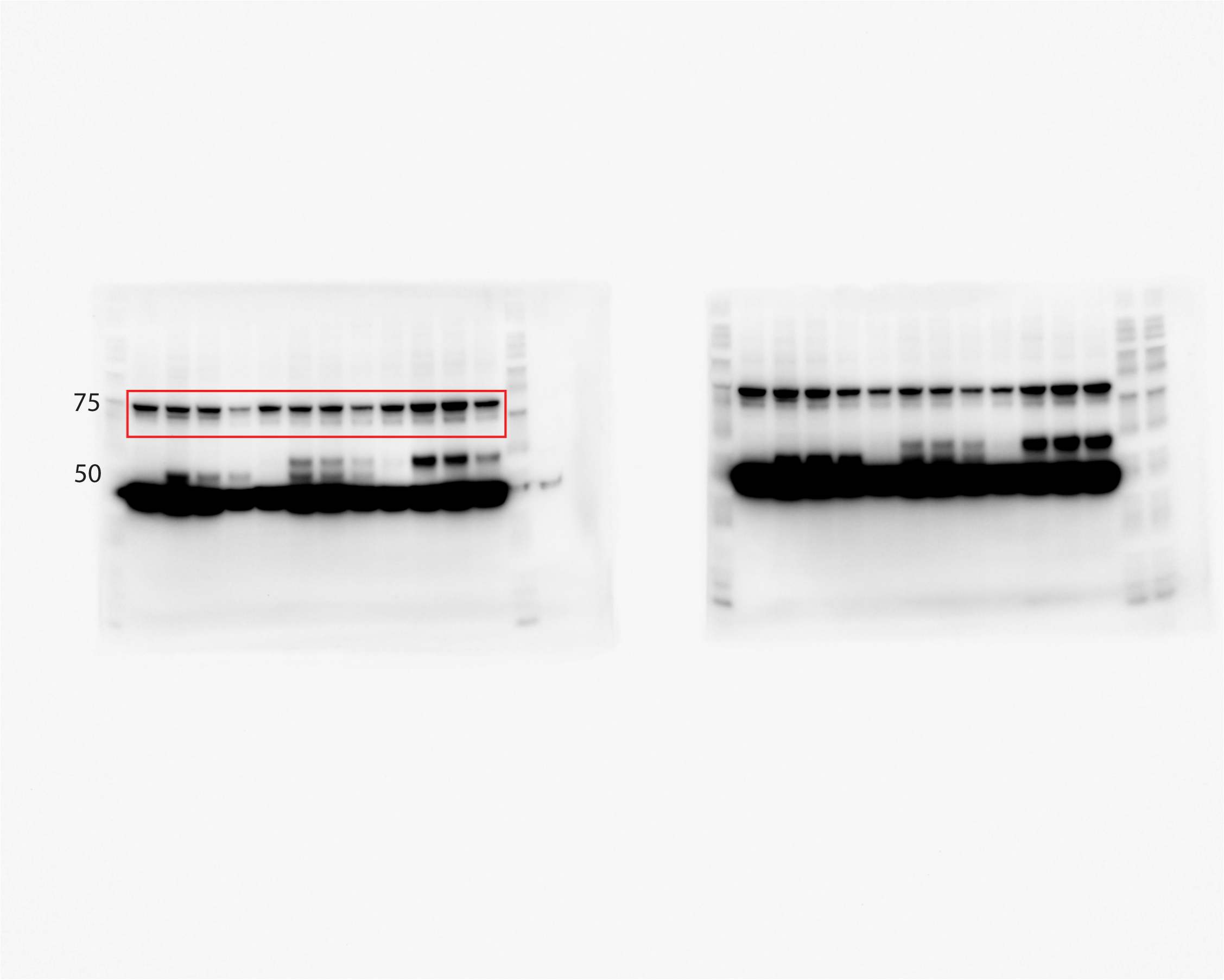

Supplement: Supplementary file 5 — Source data Fig. 2 [file 44318_2024_189_MOESM5_ESM.zip › Figure 2/2C/HSP70.tif]

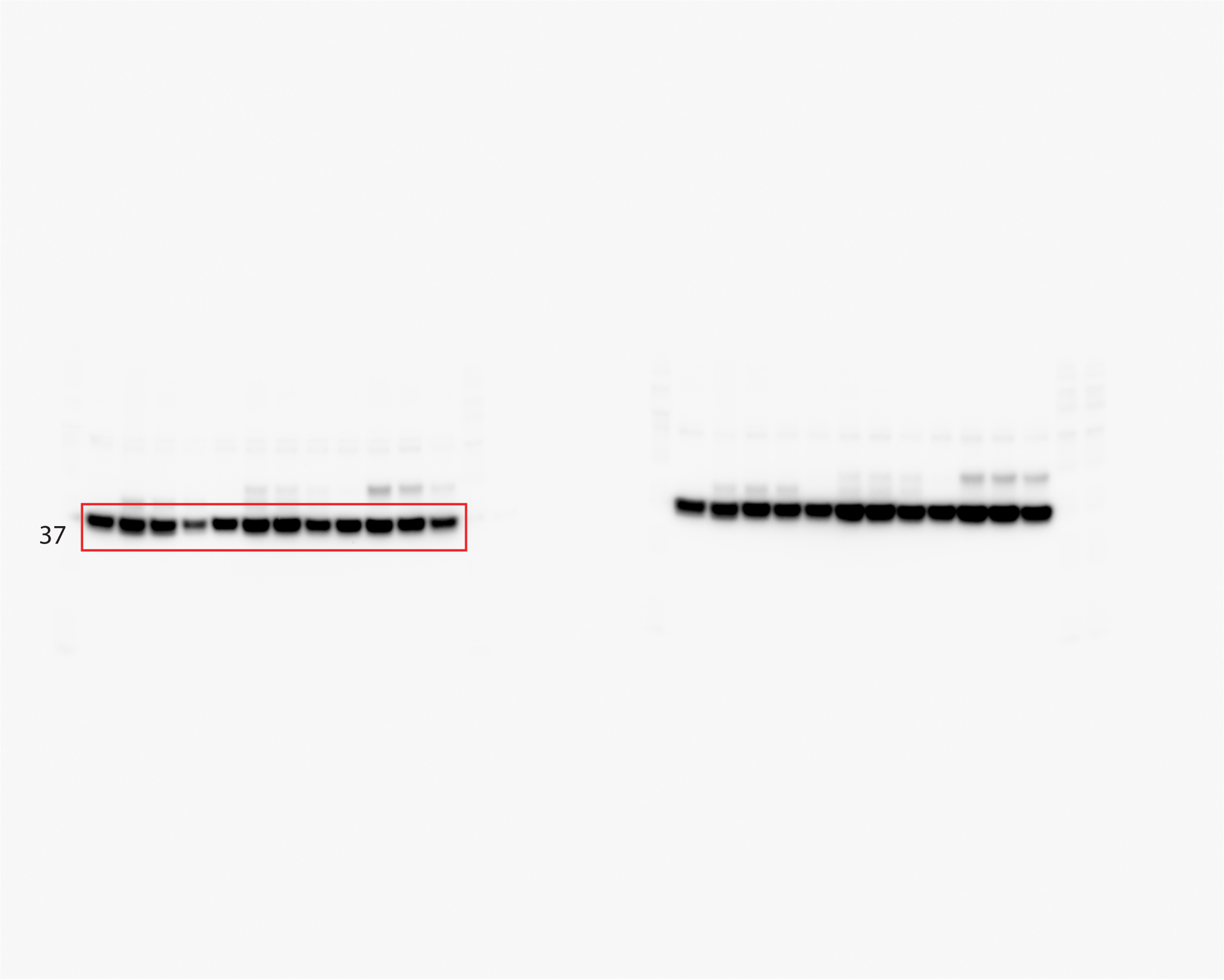

Supplement: Supplementary file 5 — Source data Fig. 2 [file 44318_2024_189_MOESM5_ESM.zip › Figure 2/2C/actin.tif]

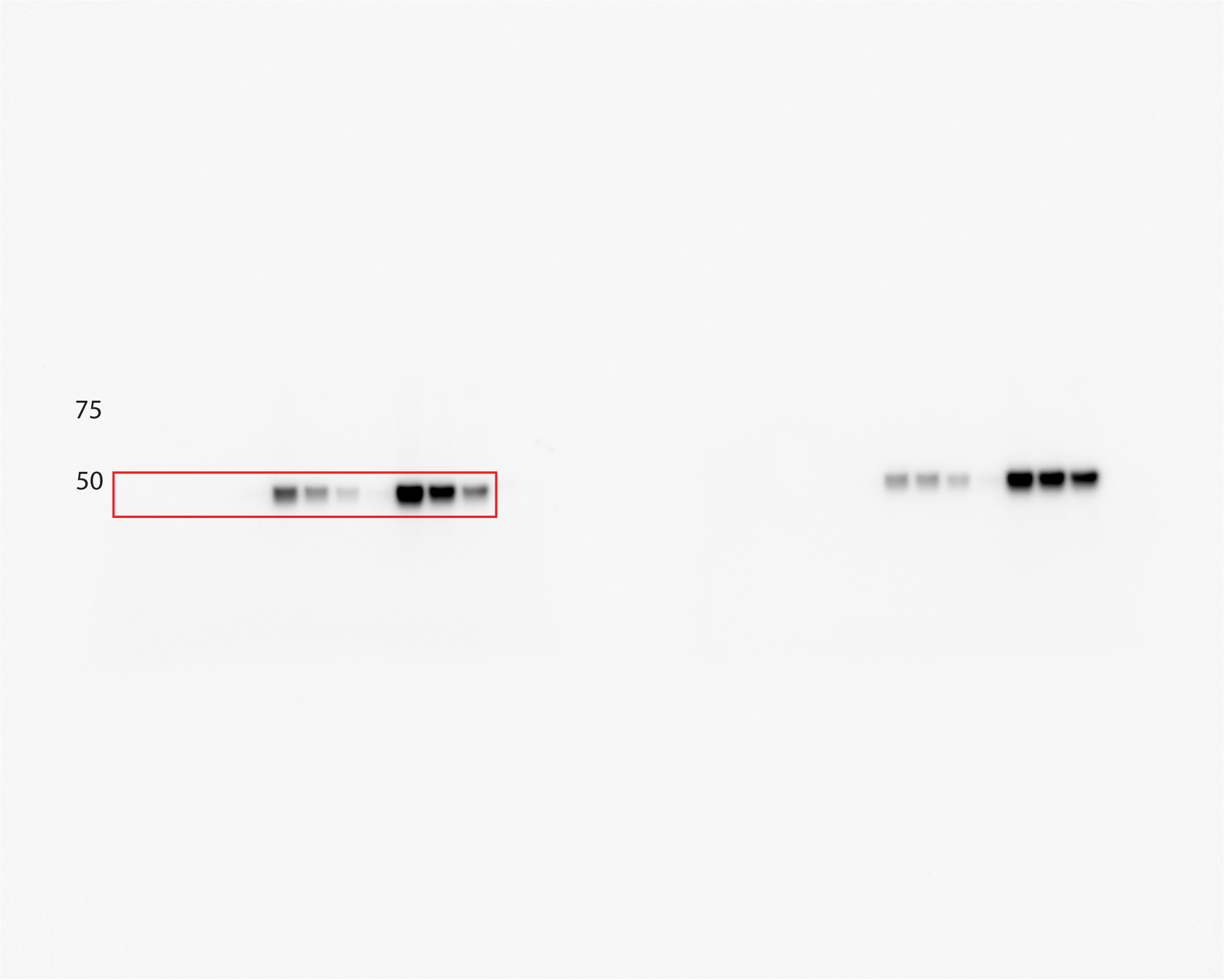

Supplement: Supplementary file 5 — Source data Fig. 2 [file 44318_2024_189_MOESM5_ESM.zip › Figure 2/2C/FLAG.tif]

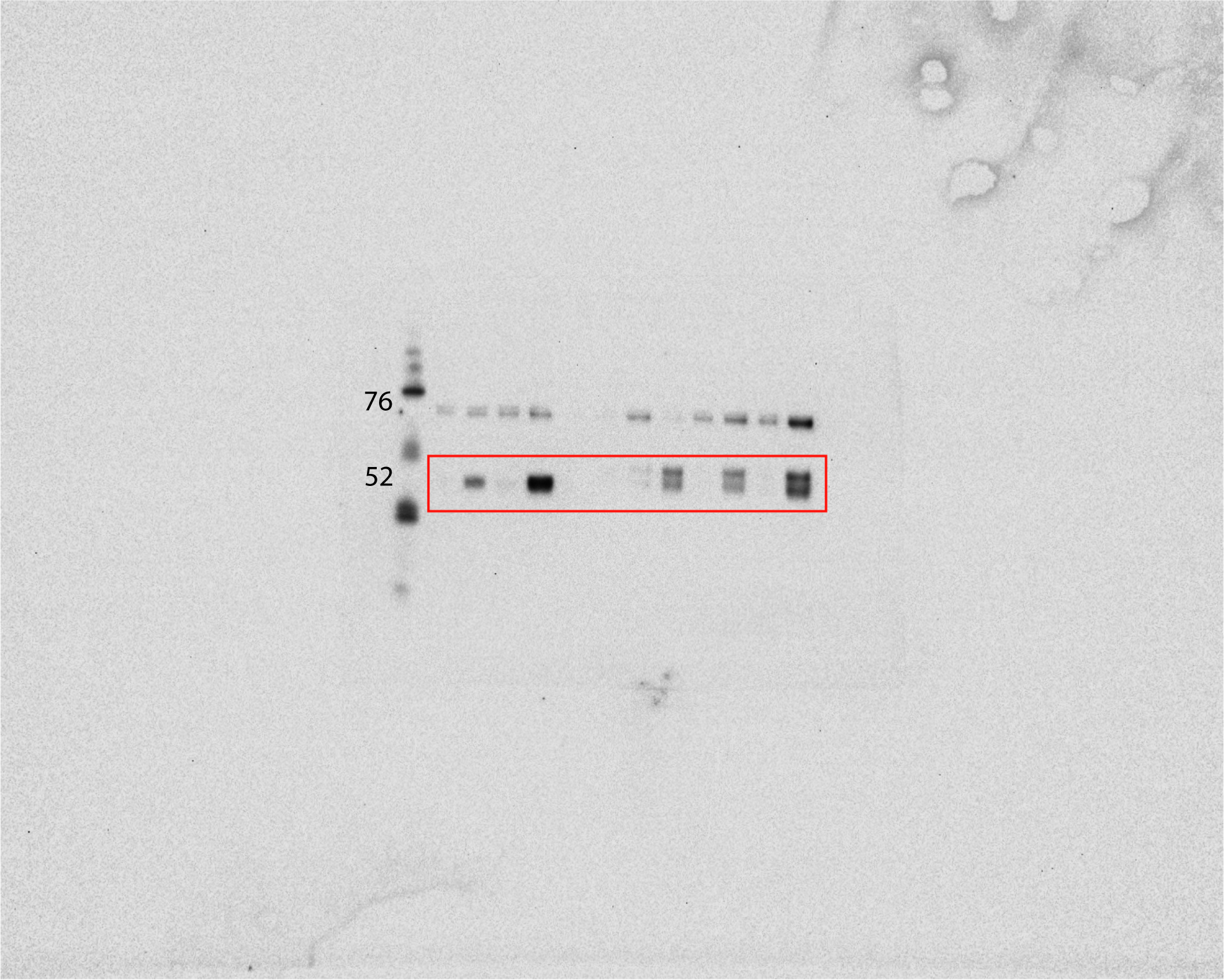

Supplement: Supplementary file 5 — Source data Fig. 2 [file 44318_2024_189_MOESM5_ESM.zip › Figure 2/2B/TRP53.tif]

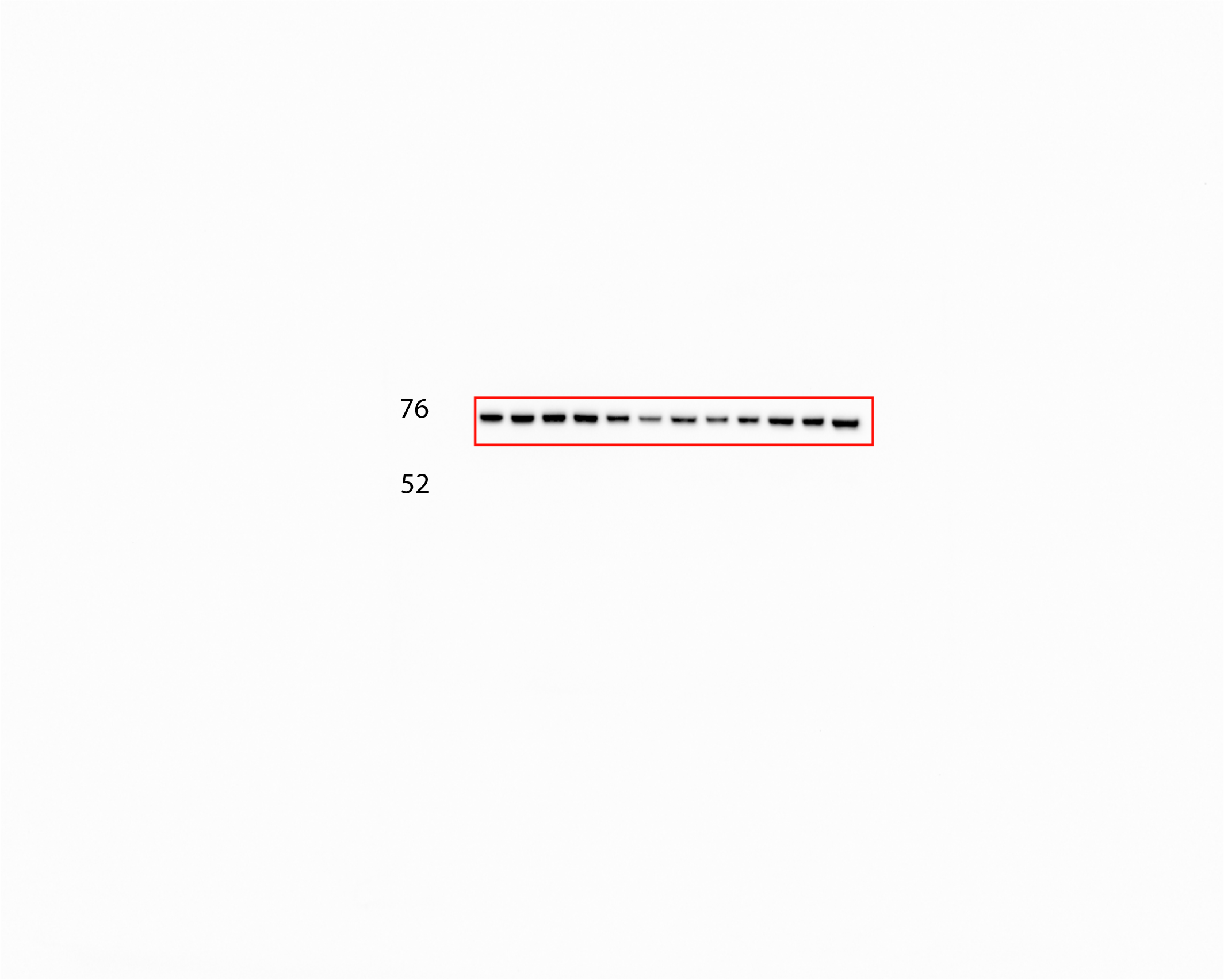

Supplement: Supplementary file 5 — Source data Fig. 2 [file 44318_2024_189_MOESM5_ESM.zip › Figure 2/2B/HSP70.tif]

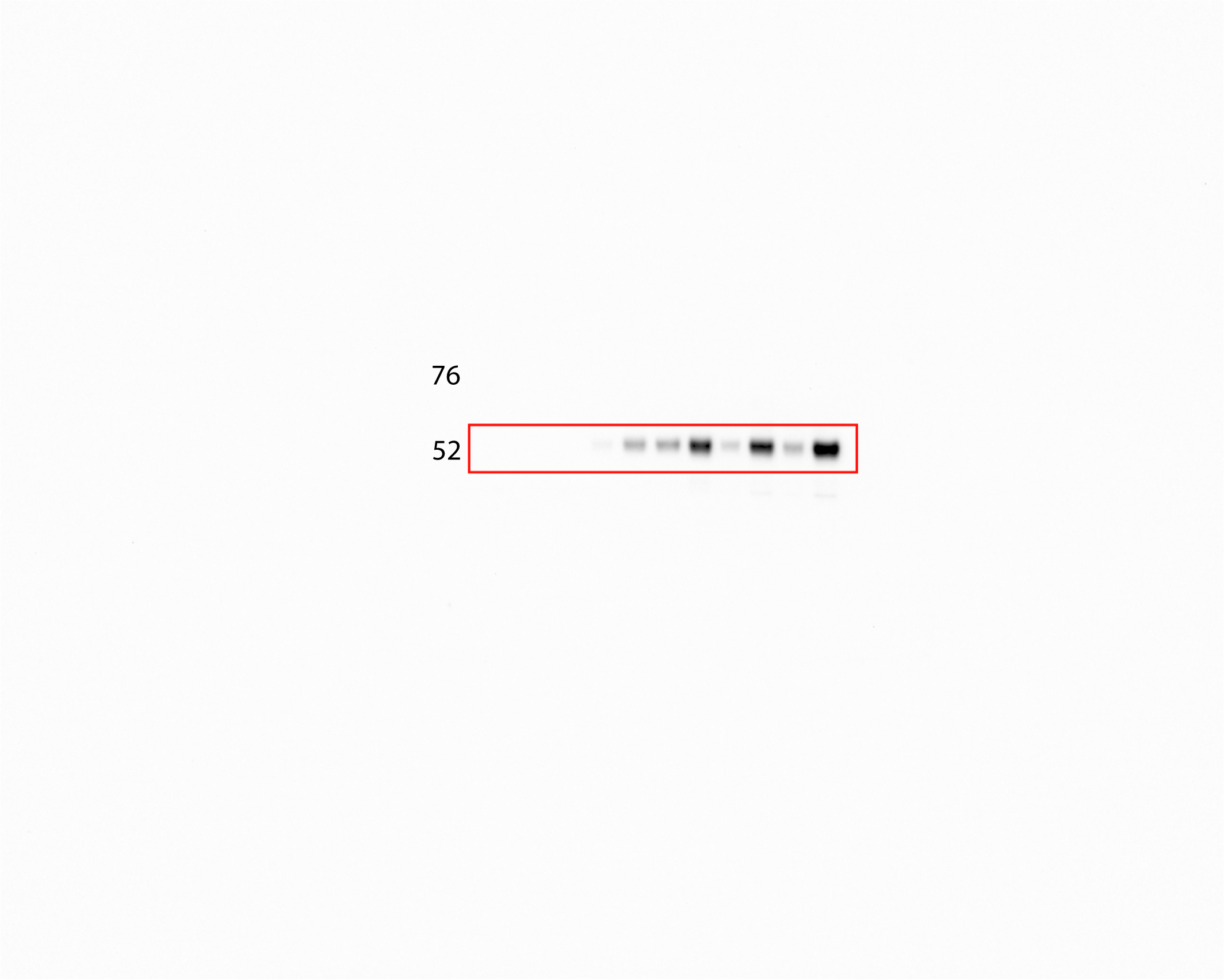

Supplement: Supplementary file 5 — Source data Fig. 2 [file 44318_2024_189_MOESM5_ESM.zip › Figure 2/2B/FLAG.tif]

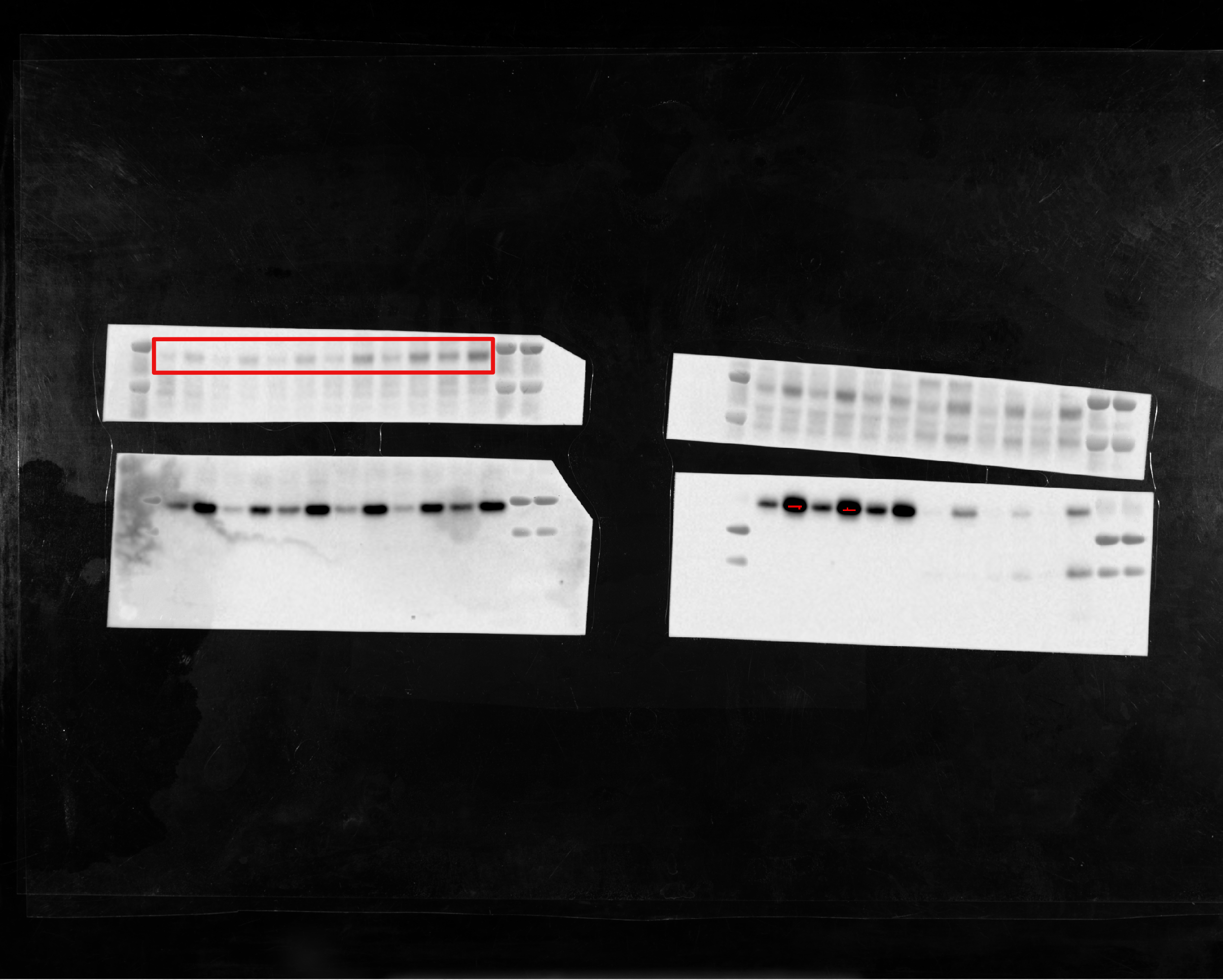

Supplement: Supplementary file 6 — Source data Fig. 3 [file 44318_2024_189_MOESM6_ESM.zip › Figure 3/3F/p21-IRES-GFP_MDF_Anti-p53.tiff]

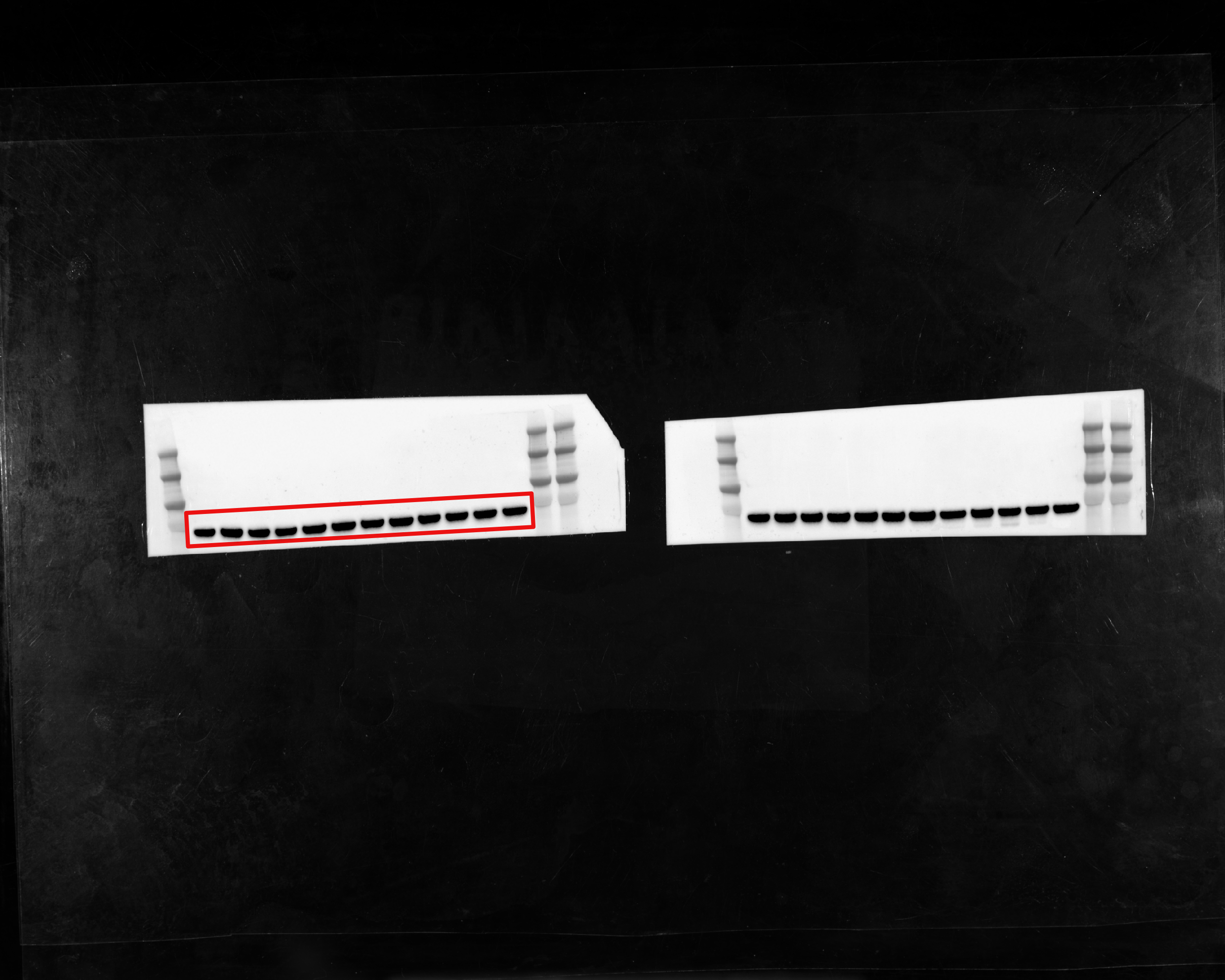

Supplement: Supplementary file 6 — Source data Fig. 3 [file 44318_2024_189_MOESM6_ESM.zip › Figure 3/3F/p21-IRES-GFP_MDF_Anti-HPS70.tiff]

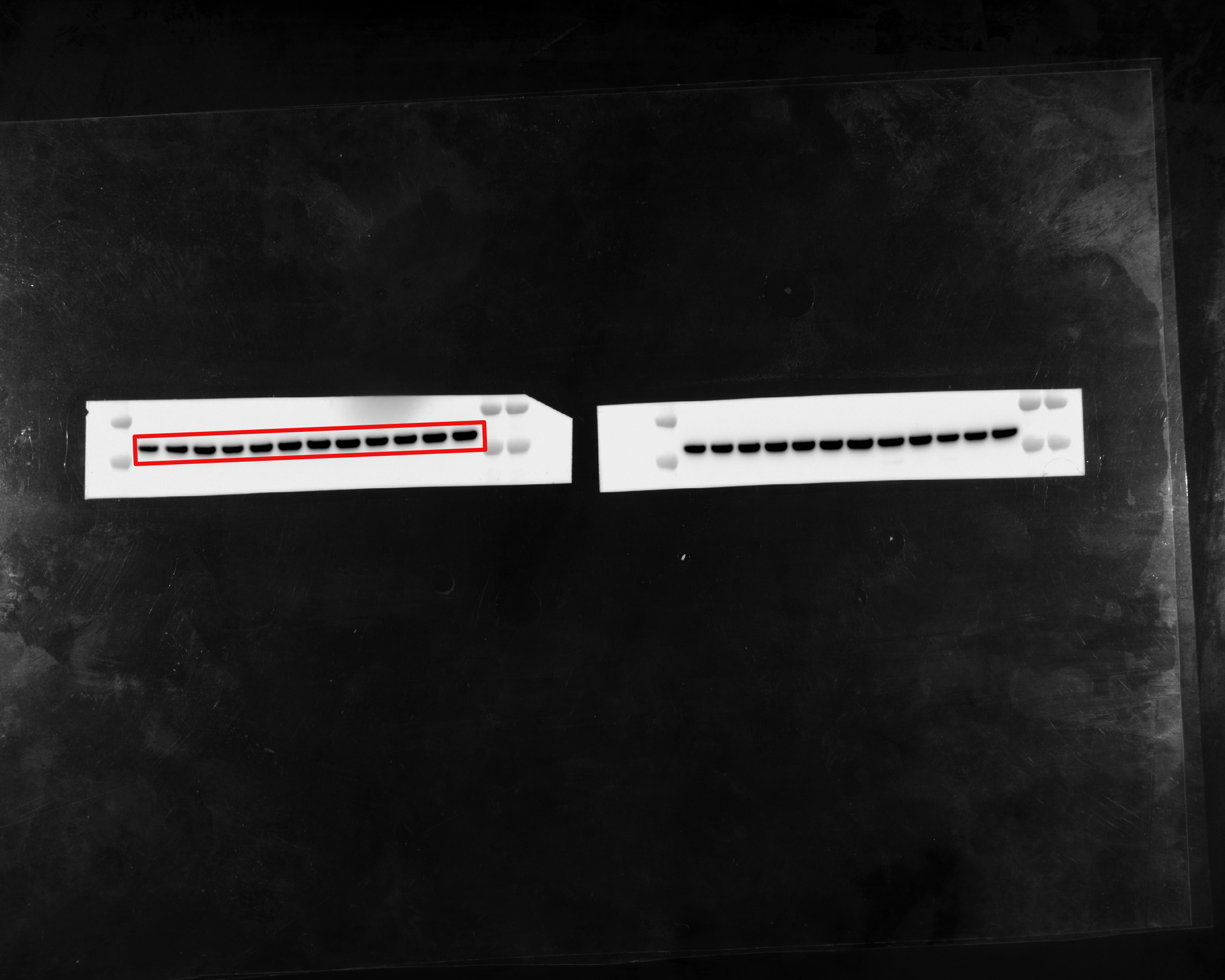

Supplement: Supplementary file 6 — Source data Fig. 3 [file 44318_2024_189_MOESM6_ESM.zip › Figure 3/3F/p21-IRES-GFP_MDF_Anti-BActin.tiff]

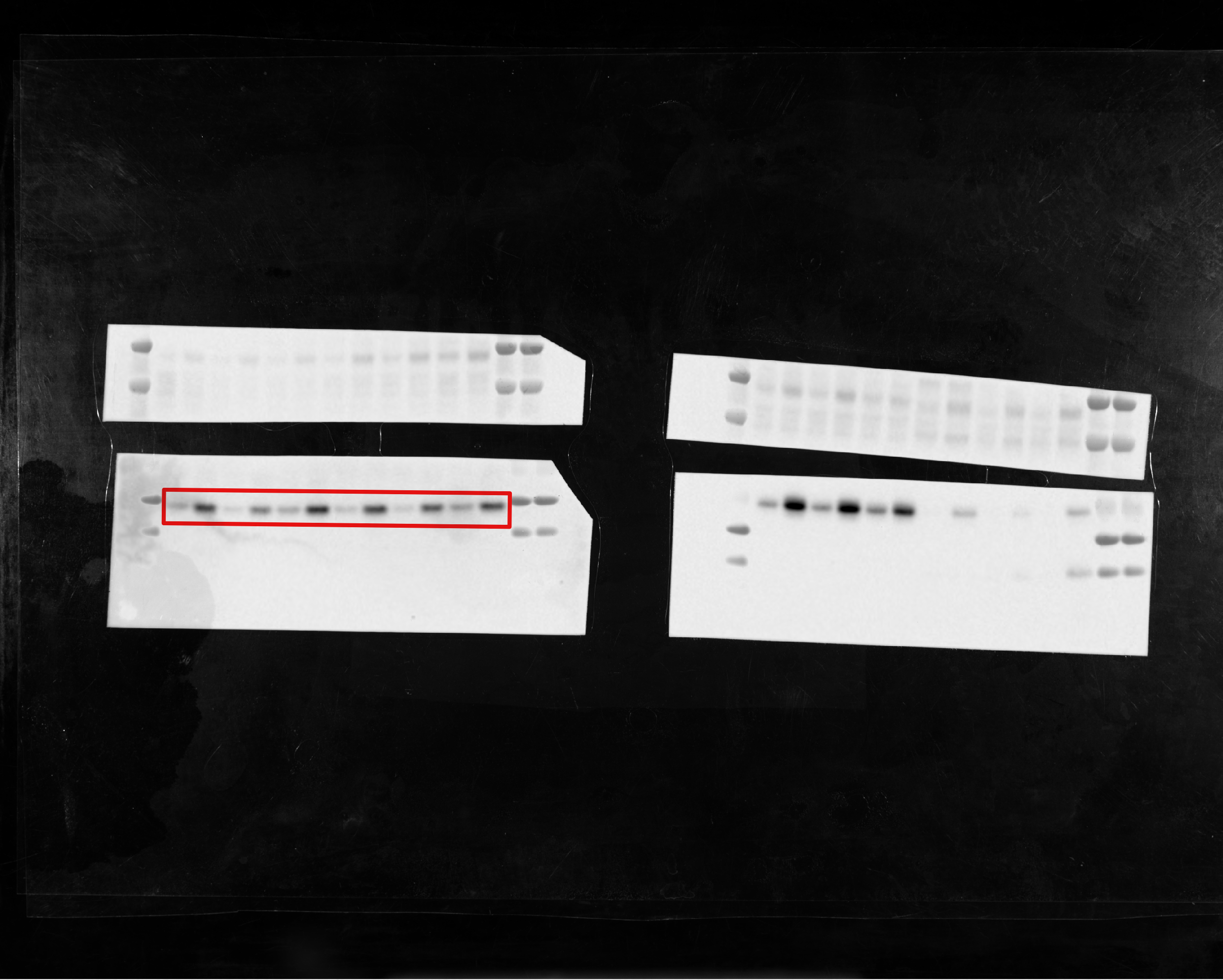

Supplement: Supplementary file 6 — Source data Fig. 3 [file 44318_2024_189_MOESM6_ESM.zip › Figure 3/3F/p21-IRES-GFP_MDF_Anti-p21.tiff]

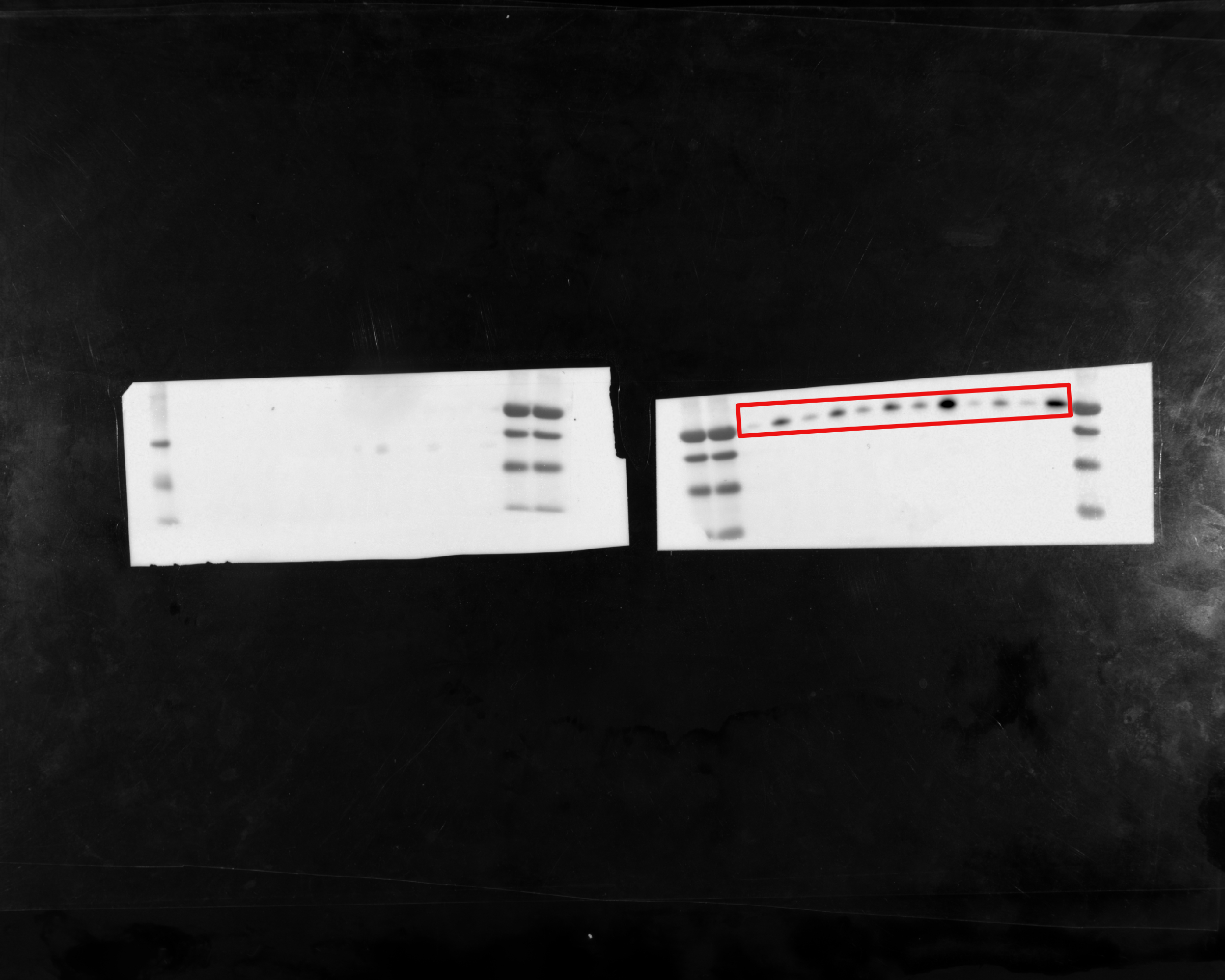

Supplement: Supplementary file 8 — Source data Fig. 6 [file 44318_2024_189_MOESM8_ESM.zip › Figure 6/6F/PUMA-tdTomato_Thymocytes_Anti-PUMA.tiff]

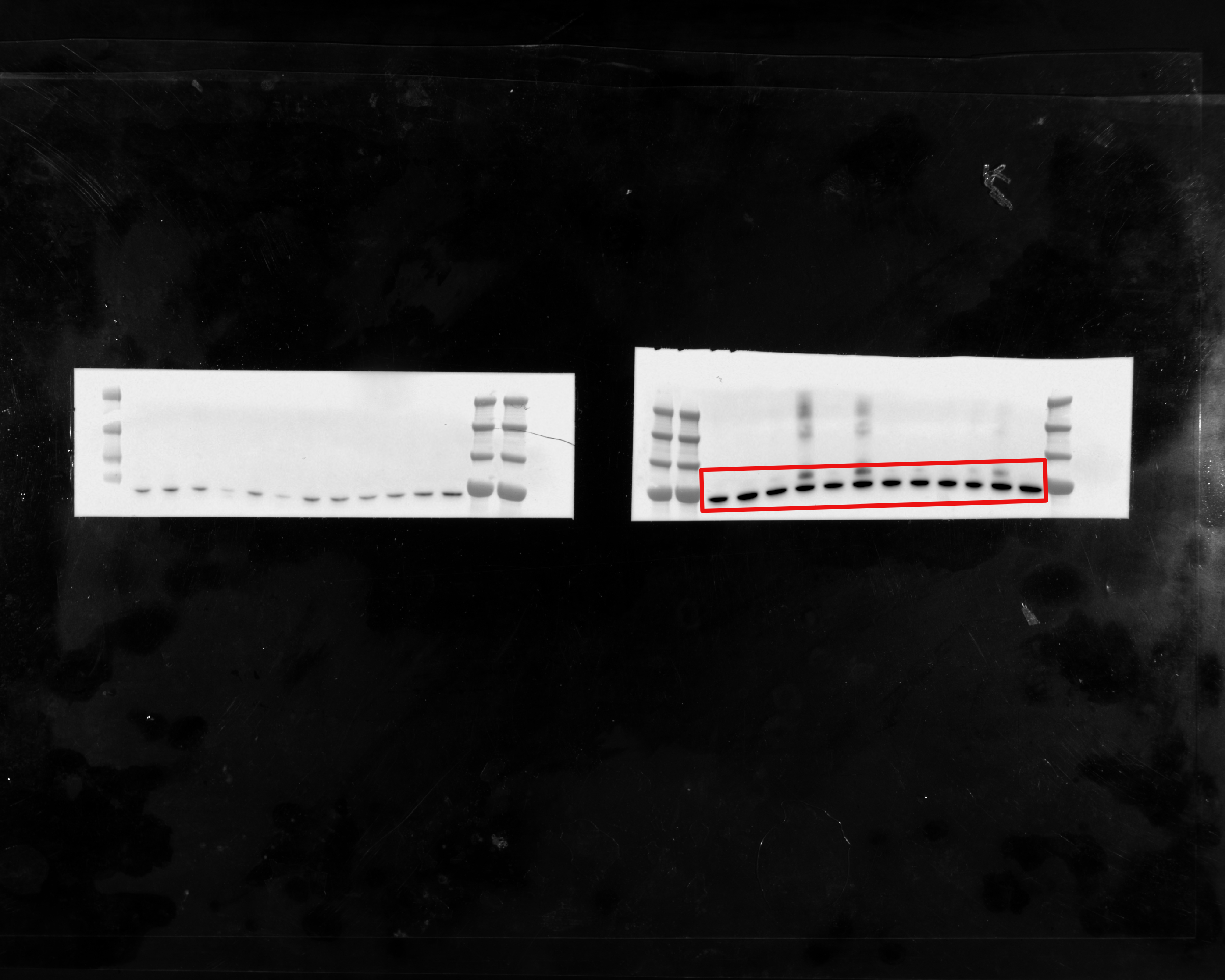

Supplement: Supplementary file 8 — Source data Fig. 6 [file 44318_2024_189_MOESM8_ESM.zip › Figure 6/6F/PUMA-tdTomato_Thymocytes_Anti-Actin.tiff]
