# Supplementary material for: Mouse models to investigate in situ cell fate decisions induced by p53
Source: EMBO J. 2024 Aug 19;43(19):12. doi: 10.1038/s44318-024-00189-z (PMC11445477; doi:10.1038/s44318-024-00189-z)
Supplement: Supplementary file 10 — Expanded View Figures [file 44318_2024_189_MOESM10_ESM.pdf]

Expanded View Figures

Figure EV1. Extra data related to *FLAG-Trp53* mice.

(**A**) Next-generation sequencing results for the inserted sequences encoding the triple-FLAG tag in the F1 generation of *FLAG-Trp53*$^{KI/+}$ mice. Each line represents the reads from 1 independent F1 mouse. A black dot indicates a matching base in the sequencing reads compared to the reference sequence. (**B**) Representative histology from aged wt, *FLAG-Trp53*$^{KI/+}$ and *FLAG-Trp53*$^{KI/KI}$ mice at the time of harvest. No tumour samples showed TRP53 staining by immunohistochemistry, indicating that they did not have mutant TRP53 driving their malignancy. Scale bar = 100 um. (**C**) RNA was extracted from thymocytes and MDFs from wt, *FLAG-Trp53*$^{KI/+}$ and *FLAG-Trp53*$^{KI/KI}$ mice that had been treated for 6 h with DMSO (vehicle control) or 1.25 Gy γ-radiation in vitro. qRT-PCR analysis was performed to determine the mRNA levels of the TRP53 target genes *Bax, Mdm2, Mlh1, Pmaip1/Noxa, Cdkn1a/p21, Bbc3/Puma* and the *Trp53* mRNA levels. Data were normalised using the ΔΔCT method, using *Hmbs* as a housekeeping gene. The data were plotted as fold-change compared to DMSO-treated samples. Data were presented as mean ± SD. $n = 4$ mice of each genotype and treatment. *p* values were calculated using a two-way ANOVA using Dunnett's correction for multiple tests. All statistical tests showed that differences were not significant (had a *p* value >0.05).

© The Author(s)

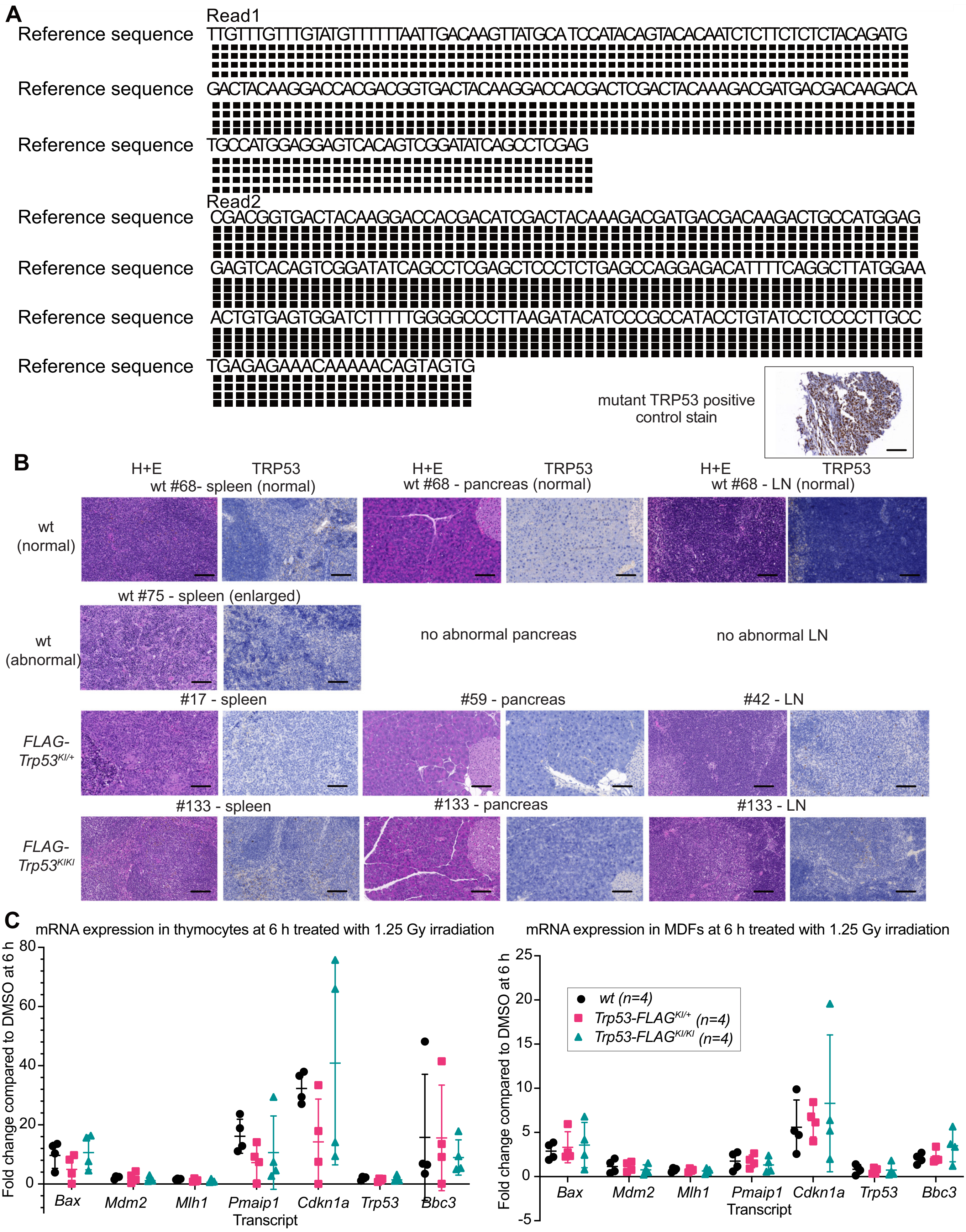
A
Read1
Reference sequence TTGTTTGTTTGTATGTTTTTTAATTGACAAGTTATGCA TCCATACAGTACACAATCTCTTCTCTCTACAGATG
Reference sequence GACTACAAGGACCACGACGGTGACTACAAGGACCACGACTCGACTACAAAGACGATGACGACAAGACA
Reference sequence TGCCATGGAGGAGTCACAGTCGGATATCAGCCTCGAG
Read2
Reference sequence CGACGGTGACTACAAGGACCACGACATCGACTACAAAGACGATGACGACAAGACTGCCATGGAG
Reference sequence GAGTCACAGTCGGATATCAGCCTCGAGCTCCCTCTGAGCCAGGAGACATTTTCAGGCTTATGGAA
Reference sequence ACTGTGAGTGGATCTTTTTGGGGCCCTTAAGATACATCCCGCCATACCTGTATCCTCCCCTTGCC
Reference sequence TGAGAGAAACAAAAACAGTAGTG
mutant TRP53 positive control stain
B
H+E
TRP53
wt #68- spleen (normal)
wt #68 - pancreas (normal)
wt #68 - LN (normal)
wt (normal)
wt #75 - spleen (enlarged)
wt (abnormal)
no abnormal pancreas
no abnormal LN
#17 - spleen
#59 - pancreas
#42 - LN
FLAG-Trp53KI/+
#133 - spleen
#133 - pancreas
#133 - LN
FLAG-Trp53KI/KI
C
mRNA expression in thymocytes at 6 h treated with 1.25 Gy irradiation
Fold change compared to DMSO at 6 h
0
20
40
60
80
Bax
Mdm2
Mlh1
Pmaip1
Cdkn1a
Trp53
Bbc3
Transcript
mRNA expression in MDFs at 6 h treated with 1.25 Gy irradiation
Fold change compared to DMSO at 6 h
-5
0
5
10
15
20
25
wt (n=4)
Trp53-FLAGKI/+ (n=4)
Trp53-FLAGKI/KI (n=4)
Bax
Mdm2
Mlh1
Pmaip1
Cdkn1a
Trp53
Bbc3
Transcript

 © The Author(s)

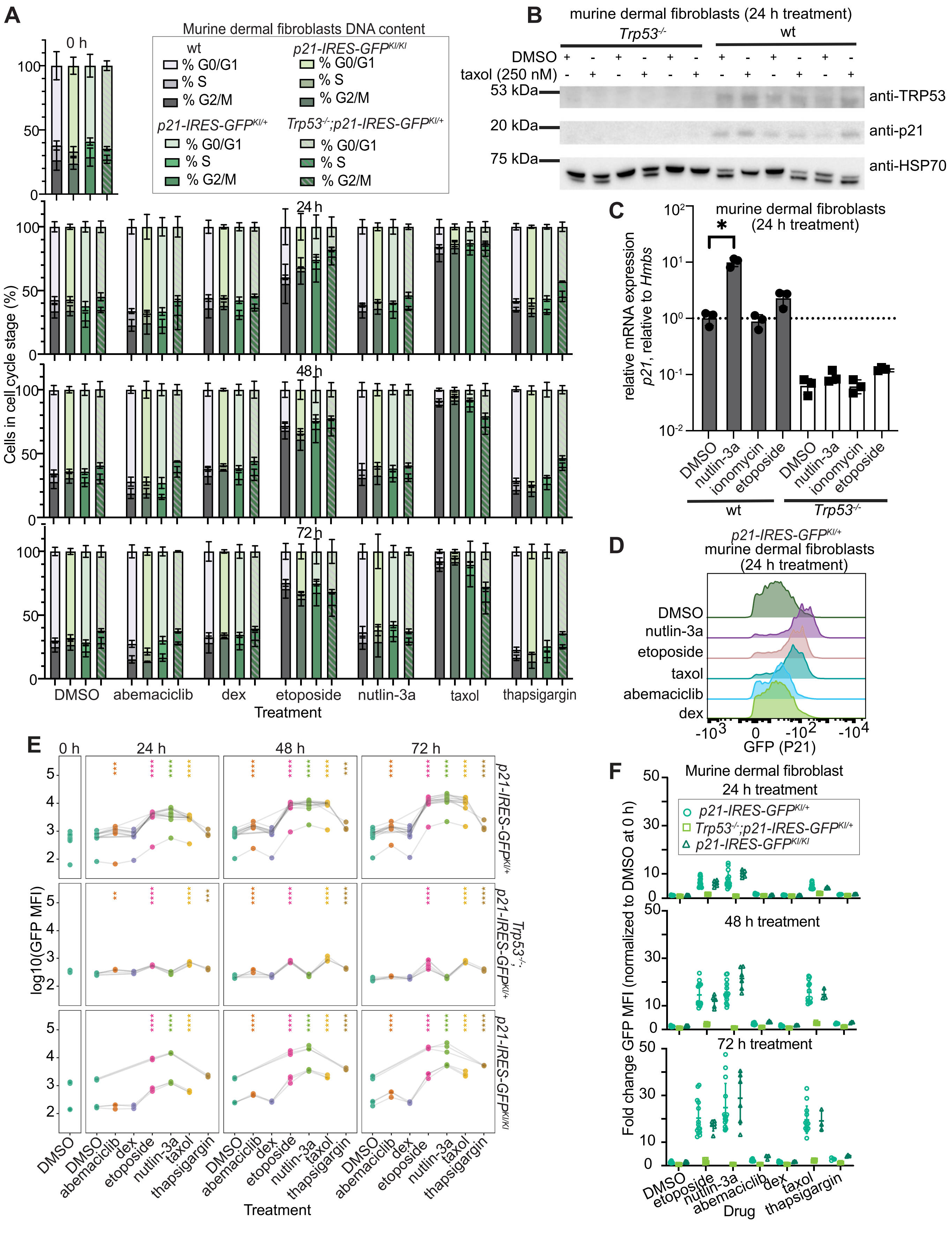
A
Murine dermal fibroblasts DNA content
wt
% G0/G1
% S
% G2/M
p21-IRES-GFP KI/KI
p21-IRES-GFP KI/+
Trp53-/-;p21-IRES-GFP KI/+
0 h
24 h
48 h
72 h
Cells in cell cycle stage (%)
DMSO
abemaciclib
dex
etoposide
nutlin-3a
taxol
thapsigargin
Treatment
B
murine dermal fibroblasts (24 h treatment)
Trp53-/-
wt
DMSO
taxol (250 nM)
53 kDa
20 kDa
75 kDa
anti-TRP53
anti-p21
anti-HSP70
C
murine dermal fibroblasts (24 h treatment)
relative mRNA expression p21, relative to Hmbs
ionomycin
D
p21-IRES-GFP KI/+ murine dermal fibroblasts (24 h treatment)
GFP (P21)
E
log10(GFP MFI)
F
Murine dermal fibroblast
24 h treatment
48 h treatment
72 h treatment
Fold change GFP MFI (normalized to DMSO at 0 h)
Drug

Figure EV2. Extra data related to *p21-IRES-GFP* mice.

(**A**) MDFs from wt, *p21-IRES-GFP*$^{KI/+}$, *p21-IRES-GFP*$^{KI/KI}$ and *Trp53*$^{-/-}$;*p21-IRES-GFP*$^{KI/+}$ mice were treated in vitro with either DMSO (vehicle control), 10 µM nutlin-3a, 1 µg/mL etoposide, 250 nM taxol, 0.5 µM dexamethasone, 5 µM abemaciclib or 1.5 µM thapsigargin. Cell cycle distribution was determined by staining with Hoechst 33342 followed by flow cytometric analysis at 0, 24, 48 and 72 h of drug treatment. The *p* values were calculated using a two-way ANOVA using Sidak's correction for multiple tests and displayed in Appendix Table S2. Data were presented as mean ± SD. $n = 3$–15 mice per genotype and treatment. (**B**) Western blot analysis for TRP53 and p21 protein levels in MDFs from wt or *Trp53*$^{-/-}$ mice 24 h after treatment with either DMSO (vehicle control) or 250 nM taxol. Probing for HSP70 was used as a loading control. Created with BioRender.com. (**C**) qRT-PCR analysis examining the levels of *p21* mRNA in MDFs from wt and *Trp53*$^{-/-}$ mice treated with either DMSO (vehicle control), 10 µM nutlin-3a, 1 µg/mL etoposide or 1 µg/mL ionomycin. Data were normalised using the ΔΔCT method, using *Hmbs* as a housekeeping gene. The data were plotted as fold-change compared to the average of the wt DMSO-treated samples. Data were presented as mean ± SD. $n = 3$ mice of each genotype and treatment. The *p* values were calculated using a one-way ANOVA using Tukey's correction for multiple tests. *P* value* ≤0.05. (**D**) Representative flow cytometry histograms showing GFP expression in MDFs generated from *p21-IRES-GFP*$^{KI/KI}$ reporter mice after 24 h of treatment with DMSO (vehicle control), 10 µM nutlin-3a, 1 µg/mL etoposide, 250 nM taxol, 1 µM dexamethasone or 5 µM abemaciclib. The data shown were representative of MDFs from $n = 6$ mice for each genotype and treatment. (**E**) Summary plots of GFP expression in MDFs from Figs. 4B and EV2D, displayed as log-transformed raw MFI values. Samples from MDFs of the same independent mouse are connected with a line. *p* values were calculated using a linear model using Sidak's correction for multiple tests. $n = 3$–14 for each genotype of mice and treatment. *P* value ** ≤0.01, *** ≤0.001, **** ≤0.0001. (**F**) Summary plots of GFP expression in MDFs from Figs. 4B and EV2D, displayed as fold-change relative to treatment with DMSO (control) at 0 h. Data displayed as mean ± SD from $n = 3$–14 cultures for each genotype of mice and treatment.

 © The Author(s)

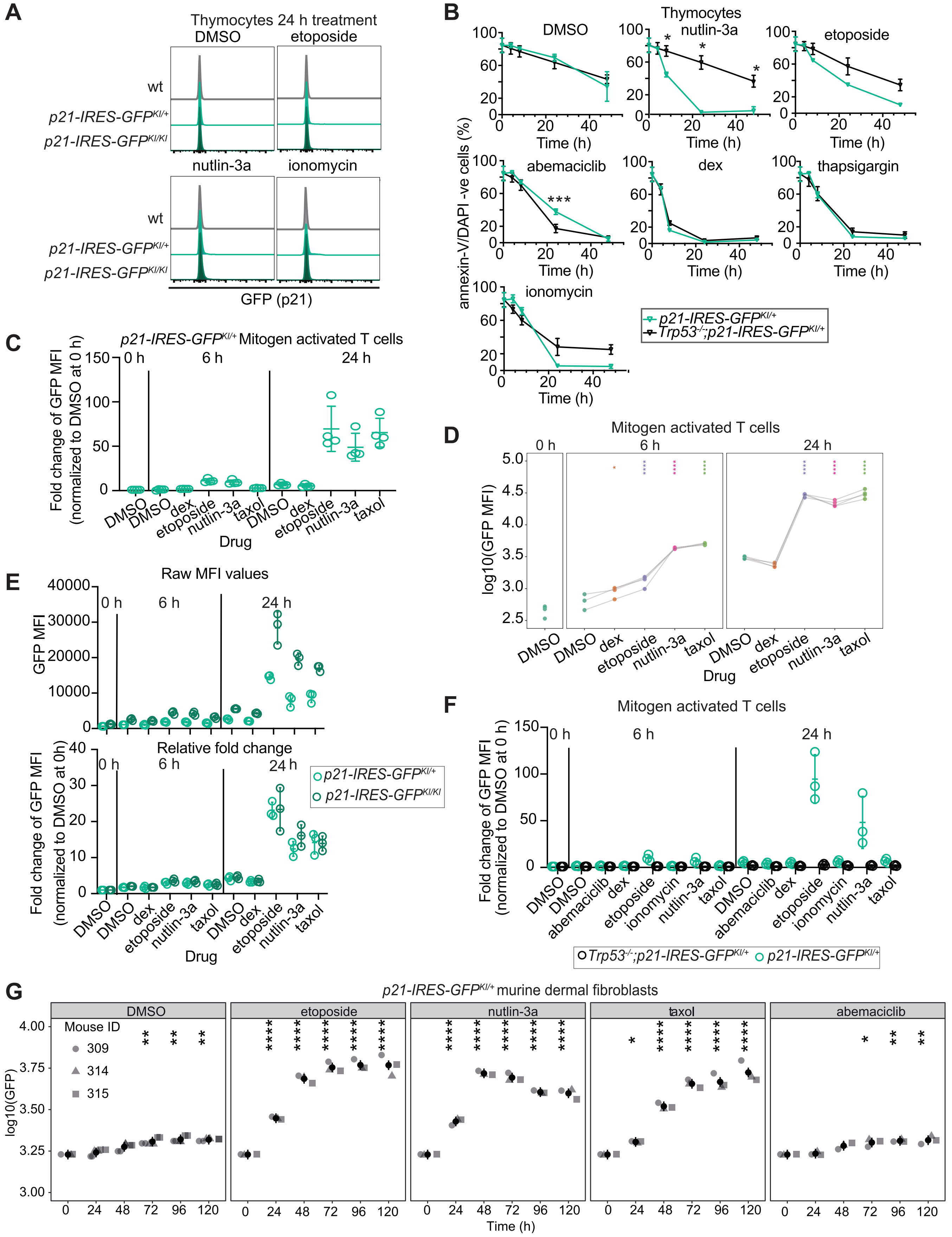

A
Thymocytes 24 h treatment
DMSO
etoposide
nutlin-3a
ionomycin
wt
p21-IRES-GFPKI/+
p21-IRES-GFPKI/KI
GFP (p21)
B
Thymocytes
DMSO
nutlin-3a
etoposide
abemaciclib
dex
thapsigargin
ionomycin
annexin-V/DAPI -ve cells (%)
Time (h)
p21-IRES-GFPKI/+
Trp53-/-;p21-IRES-GFPKI/+
C
p21-IRES-GFPKI/+ Mitogen activated T cells
Fold change of GFP MFI (normalized to DMSO at 0 h)
0 h
6 h
24 h
Drug
D
Mitogen activated T cells
log10(GFP MFI)
Drug
E
Raw MFI values
GFP MFI
Relative fold change
Fold change of GFP MFI (normalized to DMSO at 0h)
F
Mitogen activated T cells
Fold change of GFP MFI (normalized to DMSO at 0 h)
G
p21-IRES-GFPKI/+ murine dermal fibroblasts
Mouse ID
309
314
315
log10(GFP)
taxol
abemaciclib
Time (h)

Figure EV3. Thymocytes from *p21-IRES-GFP* mice do not express GFP but mitogen-activated T cells do express the *p21-IRES-GFP* reporter.

(**A**) Flow cytometry histograms showing the levels of GFP in thymocytes from a *p21-IRES-GFP*$^{KI/+}$, a *p21-IRES-GFP*$^{KI/KI}$ and a wt control mouse, that had been treated in vitro for 24 h with DMSO (vehicle control), 10 μM nutlin-3a, 1 μg/mL etoposide or 1 μg/mL ionomycin in the presence of the broad-spectrum caspase inhibitor QVD-oPH to prevent cell demolition due to apoptosis. Representative histograms from $n = 3$–7 independent thymocyte cultures per genotype and treatment are shown. (**B**) Thymocytes from *p21-IRES-GFP*$^{KI/+}$ and *Trp53*$^{-/-}$;*p21-IRES-GFP*$^{KI/+}$ mice were treated for 48 h in vitro with either DMSO (vehicle control), 10 μM nutlin-3a, 1 μg/mL etoposide, 1 μg/mL ionomycin, 0.5 μM abemaciclib, 1 μM dexamethasone or 50 nM thapsigargin. The percentages of live thymocytes (annexin-V/DAPI double negative) were determined by flow cytometric analysis. *p* values were calculated using a two-way ANOVA using Sidak's correction for multiple tests. *p* value* ≤0.05, *** ≤0.001. Data were presented as mean ± SD. $n = 3$–6 mice for each genotype and treatment. (**C**) Summary plots of GFP expression in mitogen-activated T cells from Fig. 4C, displayed as fold-change relative to treatment with DMSO (control) at 0 h. Data displayed as mean ± SD from $n = 3$–4 cultures for each genotype of mice and treatment. (**D**) Summary plots of GFP expression in mitogen-activated T cells from Fig. 4C, displayed as log-transformed raw MFI values. Samples from the same mouse are connected with a line. *p* values were calculated using a linear model using Sidak's correction for multiple tests. *p* value* ≤0.05, ****≤ 0.0001. $n = 3$–4 cultures for each genotype of mice and treatment. (**E**) Summary data of GFP expression in mitogen-activated T cells from *p21-IRES-GFP*$^{KI/+}$ and *p21-IRES-GFP*$^{KI/KI}$ mice after treatment with the indicated drugs for the indicated time showing raw mean fluorescence intensity (MFI) values compared to fold-change normalised to untreated cells at 0 h. Data displayed as mean ± SD from $n = 3$ mice for each genotype and treatment. (**F**) Summary data of GFP expression in mitogen-activated T cells from *p21-IRES-GFP*$^{KI/+}$ and *Trp53*$^{-/-}$;*p21-IRES-GFP*$^{KI/+}$ mice treated in vitro for 6 or 24 h with DMSO (vehicle control), 10 μM nutlin-3a, 1 μg/mL etoposide, 0.5 μM abemaciclib, 1 μM dexamethasone, 250 nM taxol or 1 μg/mL ionomycin displayed as fold-change normalised to untreated cells at 0 h. Data displayed as mean ± SD from $n = 3$ for each genotype of mice and treatment. (**G**) Summary plots of GFP expression in MDFs from Fig. 4E, displayed as log-transformed raw MFI values. Black symbol indicates mean, grey symbols are individual cultures. *p* values were calculated using a linear model using Sidak's correction for multiple tests. *p* value* ≤0.05, **≤0.01, ****≤0.0001. $n = 3$ cultures for each genotype of mice and treatment.

 © The Author(s)

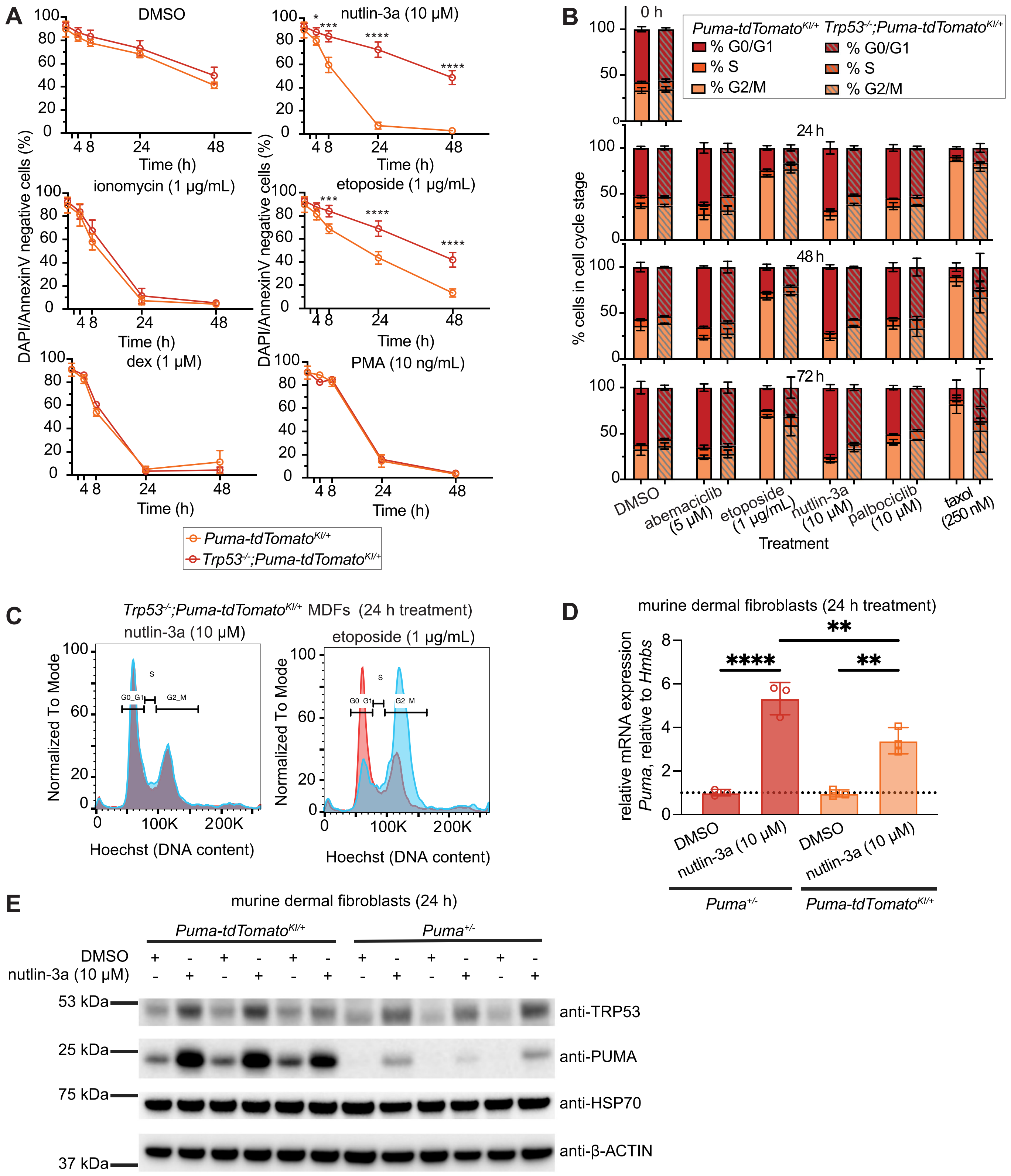
A
DMSO
nutlin-3a (10 µM)
ionomycin (1 µg/mL)
etoposide (1 µg/mL)
dex (1 µM)
PMA (10 ng/mL)
DAPI/AnnexinV negative cells (%)
Time (h)
Puma-tdTomato^KI/+
Trp53^-/-;Puma-tdTomato^KI/+
B
0 h
24 h
48 h
72 h
% cells in cell cycle stage
% G0/G1
% S
% G2/M
DMSO
abemaciclib (5 µM)
etoposide (1 µg/mL)
nutlin-3a (10 µM)
palbociclib (10 µM)
taxol (250 nM)
Treatment
C
Trp53^-/-;Puma-tdTomato^KI/+ MDFs (24 h treatment)
nutlin-3a (10 µM)
etoposide (1 µg/mL)
Normalized To Mode
G0_G1
S
G2_M
Hoechst (DNA content)
D
murine dermal fibroblasts (24 h treatment)
relative mRNA expression Puma, relative to Hmbs
DMSO
nutlin-3a (10 µM)
Puma^+/-
Puma-tdTomato^KI/+
E
murine dermal fibroblasts (24 h)
Puma-tdTomato^KI/+
Puma^+/-
DMSO
nutlin-3a (10 µM)
53 kDa
25 kDa
75 kDa
37 kDa
anti-TRP53
anti-PUMA
anti-HSP70
anti-β-ACTIN

Figure EV4. Extra data related to *Puma-tdTomato* mice.

(**A**) Thymocytes were extracted from *Puma-tdTomato*$^{KI/+}$ mice and *Trp53*$^{-/-}$;*Puma-tdTomato*$^{KI/+}$ mice, and treated for 48 h in vitro with either DMSO (vehicle control), 10 μM nutlin-3a, 1 μg/mL etoposide, 1 μg/mL ionomycin, 1 μM dexamethasone or 10 ng/mL PMA. The percentages of live thymocytes (annexin-V/DAPI double negative) were determined by flow cytometric analysis. *p* values were calculated using a two-way ANOVA using Sidak's correction for multiple tests. *p* value* ≤0.05, *** ≤0.001, **** ≤0.0001. Data were presented as mean ± SD. $n = 3$–7 for mice of each genotype and treatment. (**B**) MDFs from *Puma-tdTomato*$^{KI/+}$ and *Trp53*$^{-/-}$;*Puma-tdTomato*$^{KI/+}$ mice were treated for 72 h with either DMSO (vehicle control), 10 μM nutlin-3a, 1 μg/mL etoposide, 250 nM taxol, 10 μM palbociclib or 5 μM abemaciclib. Cell cycle distribution was determined by flow cytometric analysis after staining with Hoechst 33342 at the start of treatment (0 h), and at 24, 48 and 72 h of drug treatment. *p* values were calculated using a two-way ANOVA using Sidak's correction for multiple tests and displayed in Appendix Table S3. Data were presented as mean ± SD. $n = 3$ mice for each genotype and treatment. (**C**) Representative flow cytometry histograms of cell cycle distribution of MDFs from *Trp53*$^{-/-}$;*Puma-tdTomato*$^{KI/+}$ mice following in vitro treatment for 24 h with DMSO (vehicle control; in red), 10 μM nutlin-3a or 1 μg/mL etoposide (overlaid in blue). Cell cycle distribution was determined by flow cytometric analysis after staining with Hoechst 33342. The data shown were representative of $n = 3$ for mice of each genotype and treatment. (**D**) qRT-PCR analysis examining the levels of *Puma* mRNA in MDFs from *Puma-tdTomato*$^{KI/+}$ and *Puma*$^{+/-}$ mice treated with either DMSO (vehicle control) or 10 μM nutlin-3a for 24 h. Data were normalised using the ΔΔCT method, using *Hmbs* as a housekeeping gene. Data were plotted as fold-change compared to the average of the wt DMSO-treated samples. Data were presented as mean ± SD. $n = 3$ mice of each genotype and treatment. The *p* values were calculated using a one-way ANOVA using Tukey's correction for multiple tests. *p* value** ≤0.01, **** ≤0.0001. (**E**) Western blot analysis for TRP53 and PUMA protein levels in MDFs from *Puma-tdTomato*$^{KI/+}$ and *Puma*$^{+/-}$ mice 24 h after treatment with either DMSO (vehicle control) or 10 μM nutlin-3a. Probing for HSP70 and β-ACTIN were used as loading controls. Created with BioRender.com.

© The Author(s)

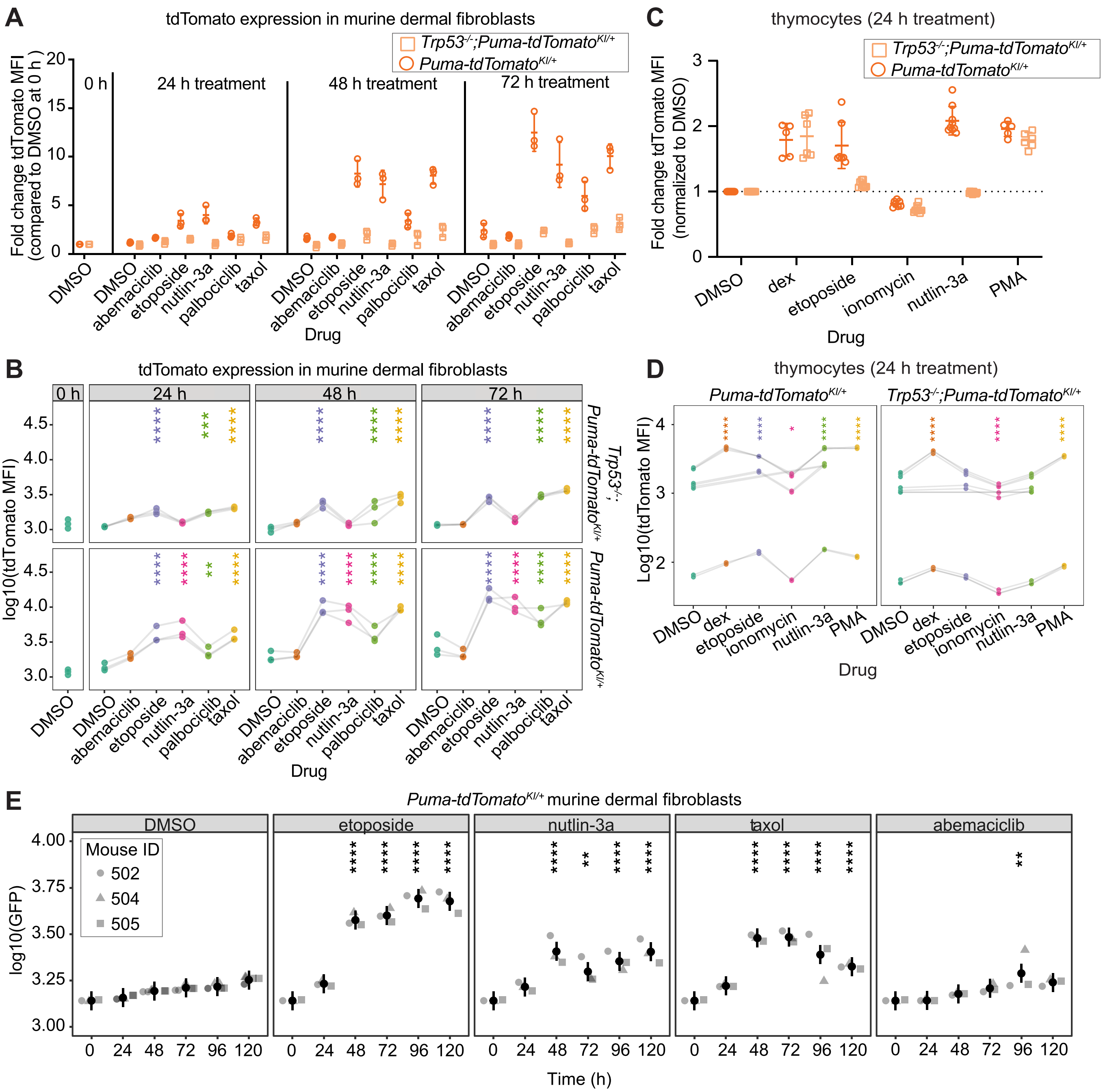

Figure EV5. Extra data related to tdTomato expression in *Puma-tdTomato* mice.

(**A**) Summary plots of tdTomato expression in MDFs from Fig. 7B, displayed as fold-change relative to treatment with DMSO (control) at 0 h. Data displayed as mean ± SD from $n = 3$ cultures for each genotype of mice and treatment. (**B**) Summary plots of tdTomato expression in MDFs from Fig. 7B, displayed as log-transformed raw MFI values. Samples from the same mouse are connected with a line. *p* values were calculated using a linear model using Sidak's correction for multiple tests. *p* value** ≤0.01, *** ≤0.001, **** ≤0.0001. $n = 3$ cultures for each genotype of mice and treatment. (**C**) Summary plots of tdTomato expression in thymocytes from Fig. 7D, displayed as fold-change relative to DMSO (control) treated samples at 0 h. Data displayed as mean ± SD from $n = 5$–10 cultures for each genotype of mice and treatment. (**D**) Summary plots of tdTomato expression in thymocytes from Fig. 7D, displayed as log-transformed raw MFI values. Samples from the same mouse are connected with a line. *p* values were calculated using a linear model using Sidak's correction for multiple tests. *p* value* ≤0.05, **** ≤0.0001. $n = 5$–10 cultures for each genotype of mice and treatment. (**E**) Summary plots of tdTomato expression in MDFs from Fig. 7E, displayed as log-transformed Raw MFI values. The black symbol indicates the mean. *p* values were calculated using a linear model using Sidak's correction for multiple tests. *p* value**≤0.01, **** ≤0.0001. $n = 3$ cultures for each genotype of mice and treatment.

© The Author(s)
